# Lifetime benefits of early detection and treatment of diabetic kidney disease

**DOI:** 10.1371/journal.pone.0217487

**Published:** 2019-05-31

**Authors:** Julia Thornton Snider, Jeffrey Sullivan, Emma van Eijndhoven, Michael K. Hansen, Nobel Bellosillo, Cheryl Neslusan, Ellen O’Brien, Ralph Riley, Seth Seabury, Bertram L. Kasiske

**Affiliations:** 1 Precision Health Economics, Los Angeles, CA, United States of America; 2 Janssen Research and Development, Spring House, PA, United States of America; 3 Janssen Diagnostics, Raritan, NJ, United States of America; 4 Janssen Global Services, Raritan, NJ, United States of America; 5 Hennepin County Medical Center, Minneapolis, MN, United States of America; University of Glasgow, UNITED KINGDOM

## Abstract

**Objectives:**

Diabetic kidney disease (DKD) is a frequent complication of diabetes with potentially devastating consequences that may be prevented or delayed. This study aimed to estimate the health and economic benefit of earlier diagnosis and treatment of DKD.

**Methods:**

Life expectancy and medical spending for people with diabetes were modeled using The Health Economics Medical Innovation Simulation (THEMIS). THEMIS uses data from the Health and Retirement Study to model cohorts of individuals over age 50 to project population-level lifetime health and economic outcomes. DKD status was imputed based on diagnoses and laboratory values in the National Health and Nutrition Examination Survey. We simulated the implementation of a new biomarker identifying people with diabetes at an elevated risk of DKD and DKD patients at risk of rapid progression.

**Results:**

Compared to baseline, the prevalence of DKD declined 5.1% with a novel prognostic biomarker test, while the prevalence of diabetes with stage 5 chronic kidney disease declined 3.0%. Consequently, people with diabetes gained 0.2 years in life expectancy, while per-capita annual medical spending fell by 0.3%. The estimated cost was $12,796 per life-year gained and $25,842 per quality-adjusted life-year.

**Conclusions:**

A biomarker test that allows earlier treatment reduces DKD prevalence and slows DKD progression, thereby increasing life expectancy among people with diabetes while raising healthcare spending by less than one percent.

## Introduction

Diabetic kidney disease (DKD) is one of the most frequent complications resulting from diabetes. We define DKD as chronic kidney disease (CKD)[1] in the setting of diabetes mellitus with no other obvious cause of CKD. Diabetes is now the main cause of end stage renal disease (ESRD) in most countries around the world.[2] Further, the prevalence of DKD has risen together with the prevalence of diabetes worldwide. In 2014–2015, the prevalence of diabetes was 9.4% among the US population [3] and 8.4% among adults globally.[4] In the US, approximately 1 of 3 adults with diabetes have CKD.[5]

DKD has negative consequences on mortality risk and quality of life. Individuals with DKD face over 2.5 times the mortality risk of individuals with diabetes but no kidney disease.[6] Among individuals with diabetes and stage 5 CKD, the mortality risk is 4.5 times higher than that of individuals with diabetes and no kidney disease[7] and quality of life is reduced.[8]

Additionally, DKD is costly to patients and the health care system. Among the US population in 2011, mean healthcare expenditures of DKD patients were $8,473 higher compared to diabetic patients without CKD.[9] For US Medicare patients with diabetes enrolled in fee-for-service plans, those with CKD average $24,916 in annual healthcare spending compared to $15,718 for those without CKD. Furthermore, CKD is more costly as the disease progresses to later stages.[10]

Individuals with DKD do not always receive the current standard of care because DKD often goes undiagnosed until serious complications manifest.[11–13] A major challenge to the early diagnosis of DKD is that diabetic patients are not routinely screened for deteriorating kidney function and referred to nephrologists when needed.[14] The National Kidney Foundation recommends annual screening for DKD beginning five years post-diagnosis for type 1 diabetes patients or from diagnosis for type 2 diabetes patients.[12] Screening involves measuring the albumin to creatinine ratio in a spot urine sample and measurement of serum creatinine to estimate the glomerular filtration rate.

The lifetime health and economic burden of DKD to both individuals and society are dependent on the severity of the disease; thus, both the timing of the DKD diagnosis and subsequent management of the condition could greatly influence this burden. There is emerging data on several novel prognostic biomarkers, such as soluble Tumor Necrosis Factor Receptor 1 (sTNFR1) and CKD273, a urinary proteomic biomarker panel, which may increase the ability of providers to identify patients at high risk of developing DKD and DKD patients at high risk of rapidly progressing to stage 5 CKD.[15–17] sTNFR1 has shown increased predictive ability for ESRD compared to other biomarkers, especially in patients with diabetes.[16, 18, 19] Other novel biomarkers, such as KIM-1, B2M, and CKD273 have also demonstrated consistent, improved prediction of renal decline in diabetic patients.[17, 20] Improving the ability to diagnose patients through a reliable biomarker could potentially reduce the number of unidentified cases, lead to earlier intervention, and, ultimately, prevent or delay the progression to DKD and its complications.

In this study, we sought to better understand how the use of a novel prognostic biomarker test would affect health and economic outcomes, including life expectancy, quality of life, healthcare spending, and government spending. We used The Health Economics Medical Innovation Simulation (THEMIS), a Monte-Carlo microsimulation that projects the health and economic outcomes for individuals over age 50 for their remaining lives under different assumptions about policies, technology, and health trends. Specifically, we compared the implementation of the biomarker test to the status quo without the biomarker test.

## Methods

### Overview of the model

This study used THEMIS to model health and economic outcomes for individuals with DKD, as well as related aggregate outcomes for the US population over age 50. THEMIS is a validated dynamic microsimulation based on the Future Elderly Model[21–29] that tracks individuals over age 50 throughout their remaining lives to project their disease burdens, life expectancy, quality of life, income, and health care costs until the year 2050. In the model, next year’s health states depend on today’s health states and on a set of random health shocks that vary with individuals’ own risk factors, e.g., their age, health behaviors, and current disease conditions.

In order to track DKD-related outcomes, a new module was added to THEMIS to identify individuals at risk of developing DKD or individuals with diagnosed or undiagnosed DKD, as well as measure their health and economic outcomes. Additional detail is available in S1 Appendix, which describes the THEMIS model and S2 Appendix, which describes the application of THEMIS to this study.

### Data and outcomes

THEMIS is based on data from the Health and Retirement Study (HRS), a biennial, nationally representative, longitudinal survey of Americans over the age of 50.[30, 31] Patients are transitioned into and out of health states every two years via transition probability models that were estimated using HRS data. The transition probability models include inputs risk factors such as smoking, weight, age and education, along with lagged health and financial states. Key disease prevalence models for the incoming cohorts were estimated from the National Health Interview Survey, the principal source of disease prevalence estimates in the US.[32] To avoid limitations of the cost measures in the HRS, medical expenditures outcomes were estimated via the Medicare Current Beneficiary Survey and Medical Expenditure Panel Survey. This study also used the National Health and Nutrition Examination Survey to impute DKD status in THEMIS (details below).

The outcomes examined in this study included life expectancy from age 51, quality-adjusted life-years (QALYs) from age 51, per person annual healthcare spending, government healthcare spending (including Medicare and Medicaid), government non-healthcare spending (including Social Security retirement income, Social Security Disability Insurance, and Supplemental Security Income), and total government spending (the sum of government healthcare and non-healthcare spending). All net present value costs and benefits were discounted by 3% per year to 2015 dollars.

A schematic of THEMIS can be found in Figure A in S1 Appendix.

### DKD module

To examine DKD outcomes, we incorporated a DKD module into THEMIS. To identify both diagnosed and undiagnosed DKD, we employed NHANES, which contains laboratory measurements for a nationally representative sample, making it ideal for identifying undiagnosed disease.[33] Specifically, we developed an algorithm to identify patients with DKD in NHANES. DKD was defined as diabetes with albuminuria (albumin/creatinine ratio ≥30 mg/g), impaired estimated glomerular filtration rate (eGFR<60 mL/min/1.73 m^2^), or both. This algorithm was used to impute DKD status in the HRS. To avoid inaccuracies in imputing the 5 stages of DKD, we modeled DKD as non-stage 5 CKD versus stage 5 CKD. Specifically, receipt of dialysis or eGFR <15 mL/min/1.73 m^2^ were noted in the NHANES data and used to infer stage 5 status. The algorithm classified individuals with diabetes into five groups: no DKD, diagnosed DKD without stage 5 CKD, undiagnosed DKD without stage 5 CKD, diabetes with diagnosed stage 5 CKD, or diabetes with undiagnosed stage 5 CKD. For simplicity, we refer to individuals with diagnosed or undiagnosed DKD together as the “DKD” group. The model was estimated as a two-stage probit (1: DKD versus no DKD; 2: among DKD, stage 5 CKD versus no stage 5 CKD), and an independent probit for diagnosis status that includes an indicator for stage 5 CKD. The following covariates were included in the estimation of the models: age, sex, smoking behavior, ethnicity, body mass index (BMI) class, marital status, education, and comorbidities (hypertension, heart disease, stroke, cancer, and lung disease). To model the incidence of DKD over time, we used incidence rates from longitudinal claims data and matched prevalence rates to NHANES estimates.[34] We then simulated the prevalence and severity of DKD from 2010 to 2050 using THEMIS. We compared the predicted prevalence of DKD to projections from the literature. More details on the creation of the DKD module are provided in S2 Appendix.

We performed a literature review to find parameter estimates on how DKD affects outcomes. Estimates were extracted from studies that best fit our study population. Based on the literature, DKD was modeled to raise mortality risk by 2.68 times [6] and healthcare spending by 59%[10] relative to diabetes without DKD. Diabetes with stage 5 CKD was assumed to further raise healthcare spending by 362%[10] and mortality risk to 4.46,[7] while reducing quality of life by 0.049[8]. In addition, individuals with diabetes and stage 5 CKD were assumed to be 7.17 times more likely not to be working than the general population.[35] Based on an analysis of private healthcare claims data from 2011 to 2013, we assumed that those diagnosed with DKD would be offered currently available treatments (i.e. angiotensin-converting-enzyme inhibitors or angiotensin II receptor blockers) at an average cost of $23 per month,[34] and that their risk of DKD progression would be cut by 20% with such treatments.[36] Parameter values and underlying assumptions are provided in S2 Appendix. In addition, individuals with diabetes and stage 5 CKD were assumed to be 7.17 times more likely not to be working than the general population.[35] Based on an analysis of private healthcare claims data from 2011 to 2013, we assumed that those diagnosed with DKD would be offered currently available treatments (i.e. angiotensin-converting-enzyme inhibitors or angiotensin II receptor blockers) at an average cost of $23 per month,[34] and that their risk of DKD progression would be cut by 20% with such treatments.[36] Parameter values and underlying assumptions are provided in S2 Appendix.

### Scenario implementation

Two scenarios were examined, in addition to a simulation of the current standard of care: (i) a new biomarker test to identify patients at high risk; (ii) and that same test coupled with a more effective treatment. Under these scenarios, we compared results in medical expenditures, QALYs, survival gains, and government spending to a status quo scenario in which we assumed no intervention (i.e., the current standard of care).

Several assumptions were applied to each scenario. In the biomarker scenario, we assumed that everyone with diabetes would be tested for the biomarker every 2 years at a cost of $35.[37] We further assumed that 30% of tested individuals would have a positive biomarker result, and would be offered currently available treatments at a cost of $23 per month.[34] For individuals with a positive biomarker result, the probability of progressing to DKD was assumed to be 2.5 times greater than those with a negative result, based on emerging prognostic biomarker data.[16, 38] We also assumed that the probability of progressing from no DKD to DKD and from any-stage DKD to stage 5 DKD would be the same for model tractability. (Incidence and prevalence rates of DKD and stage 5 DKD from the model were checked against NHANES and claims data to verify the reasonableness of this assumption.) Using a novel biomarker, we assumed that the rate of DKD diagnosis would double because at-risk individuals would be monitored more closely.

We also considered two additional scenarios exploring how the effects of the biomarker test would differ when combined with more effective treatment. Specifically, we considered increasing the treatment effectiveness at slowing DKD progression by 50% or 100% over the current standard of care.

## Results

**Fig 1:**
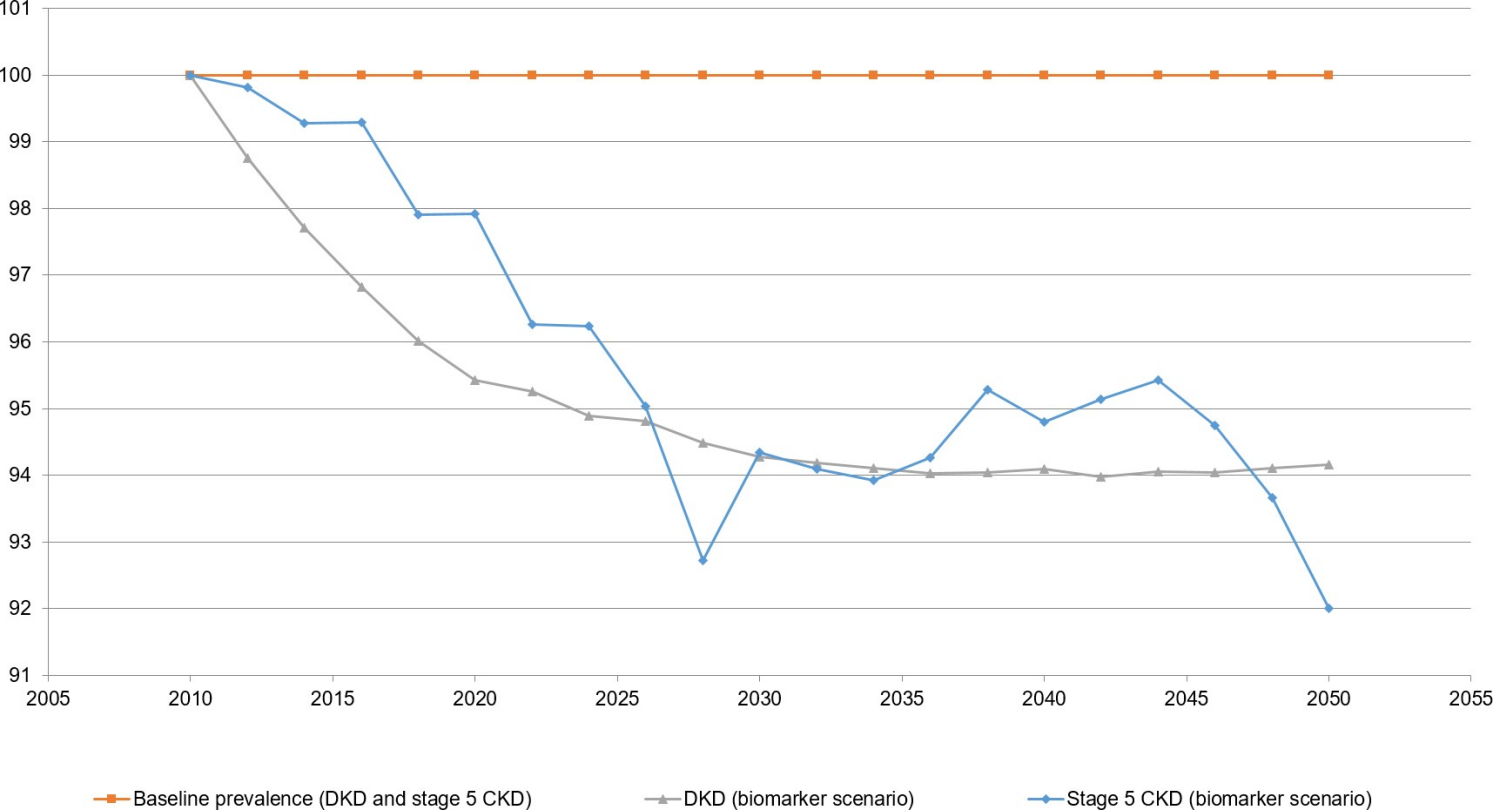
DKD and diabetes with stage 5 CKD prevalence among US population aged ≥ 51. Notes: DKD indicates diabetic kidney disease; CKD, chronic kidney disease.

Our simulation results indicate that the implementation of a novel biomarker test, used in addition to current standard of care, would reduce the prevalence of DKD and diabetes with stage 5 CKD among the US population over age 50, relative to the baseline assumption of no novel biomarker test (Fig 1). Specifically, we estimate that the prevalence of DKD would fall by 6% by 2050. Over the same period, we estimate the prevalence of diabetes with stage 5 CKD would fall by 8%. The estimates for diabetes with stage 5 CKD appear “noisier” due to the lower number of individuals with diabetes and stage 5 CKD in the underlying data the model is based on (Table A in S2 Appendix).

**Fig 2:**
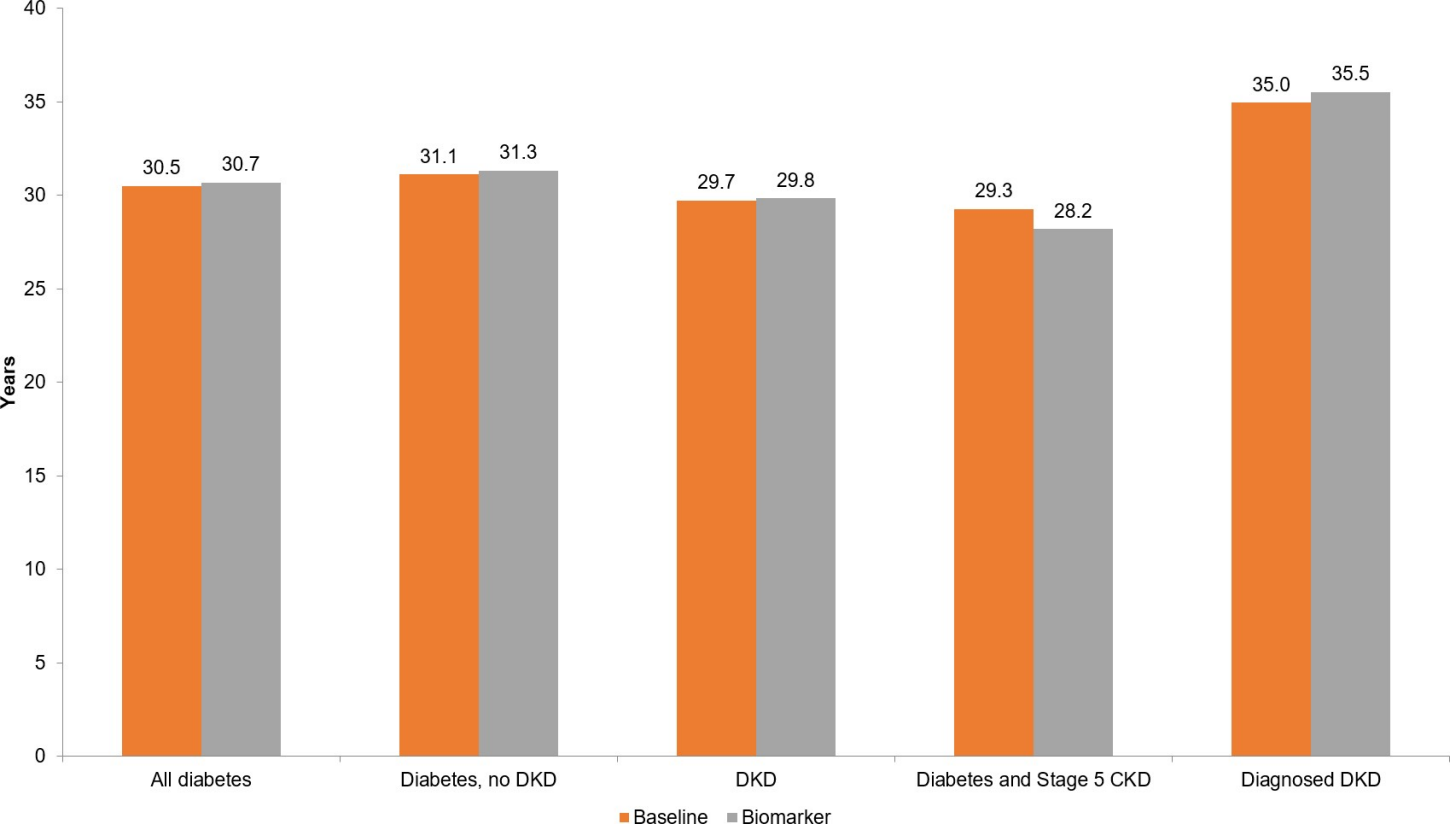
Life expectancy from age 51, by group. Notes: Reported life expectancies are the average values for a group across all years of the simulation (2010–2050). DKD indicates diabetic kidney disease; CKD, chronic kidney disease.

Because mortality risk rises when individuals with diabetes develop DKD and progress to stage 5 CKD, we estimate the implementation of the biomarker test would raise life expectancy among individuals with diabetes by identifying and treating at-risk individuals sooner (Fig 2). Specifically, looking at average life expectancy across all simulated cohorts, we estimate the life expectancy of the average individual with diabetes from age 51 would rise from 30.5 to 30.7 years, a gain of 0.2 years. Among individuals with diabetes but no DKD, we estimate a life expectancy gain from 31.1 to 31.3 years, primarily from prevented and delayed progression to DKD. Among individuals with DKD–for whom it is no longer possible to prevent or delay the development of DKD–we estimate a smaller life expectancy gain of 0.1 years, from 29.7 to 29.8 years. The estimated decline in life expectancy among people with diabetes and stage 5 CKD from 29.3 to 28.2 years may be driven by selection because less healthy people reach this stage under the biomarker scenario. Finally, we observe the highest life expectancy and highest life expectancy gain, from 35.0 to 35.5 years, among people with diagnosed DKD. Individuals must be diagnosed to receive treatment, so gains from treatment are concentrated in this group. Moreover, socioeconomically advantaged individuals, who have higher life expectancy to begin with [39], are more likely to be in this group due to their more regular contact with the healthcare system. Additionally, we estimate the QALYs of the average individual with diabetes from age 51 would increase with the implementation of the biomarker from 18.8 to 18.9 (not shown).

**Fig 3:**
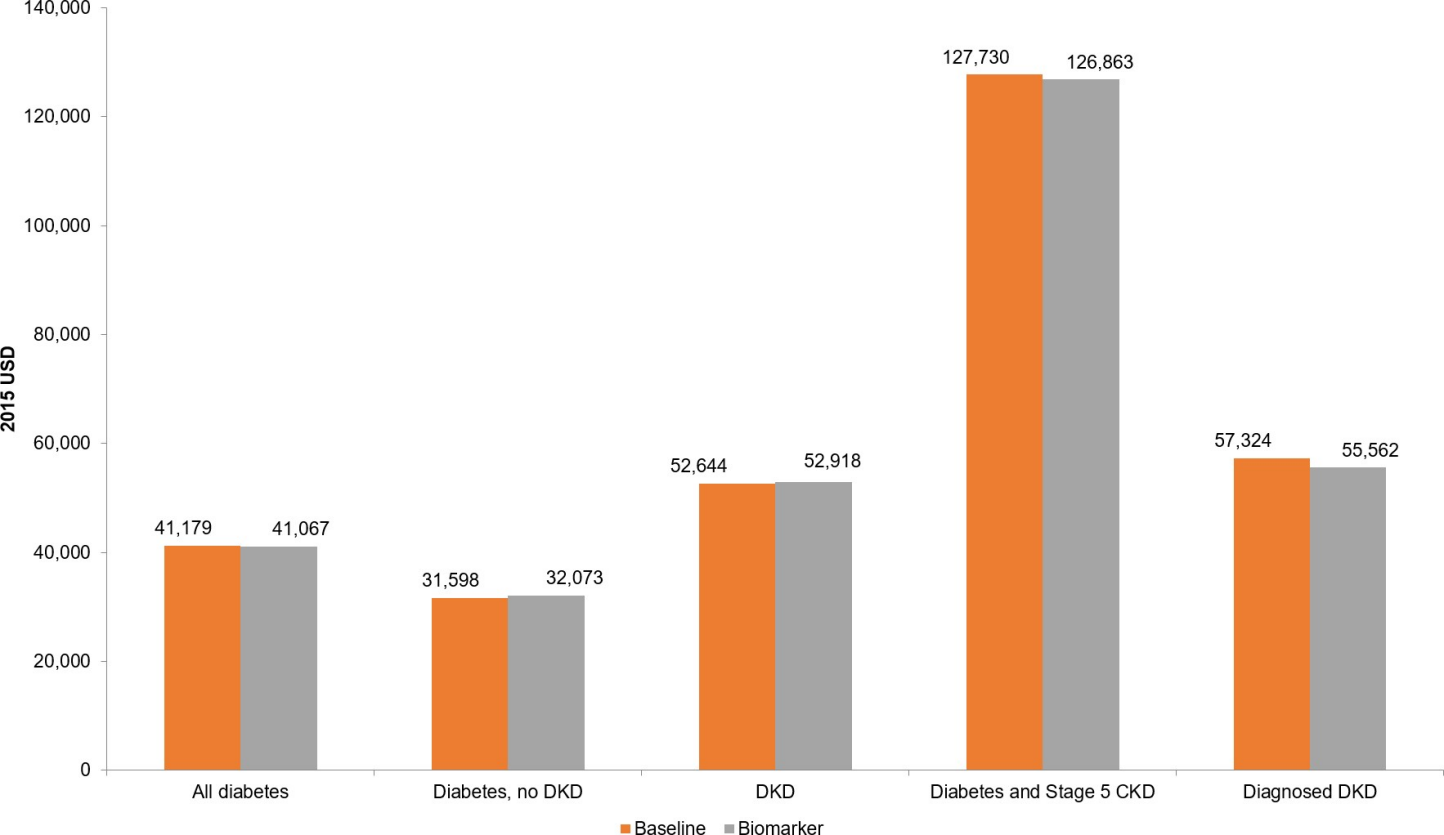
Annual healthcare expenditure per person, by group. Notes: Reported healthcare expenditures per person are the average values for a group across all years of the simulation (2010–2050). DKD indicates diabetic kidney disease; CKD, chronic kidney disease.

Examining annual per person healthcare spending (Fig 3), we estimate that the implementation of the biomarker would decrease average spending on individuals with diabetes (from $41,179 to $41,067). Dividing this group into individuals with and without DKD, we estimate both an increase in spending on individuals with diabetes but no DKD (from $31,598 to $32,073) and on individuals with DKD (from $52,644 to $52,918). This increase in spending on both subgroups and the decrease in spending on the overall group stems from the fact that the prevalence of DKD among people with diabetes would fall in the biomarker scenario. Essentially, spending on all people with diabetes is a weighted average of spending on those with and without DKD. While the spending for both subgroups rises with the biomarker, the declining prevalence of DKD implies a larger weight on the low-cost no DKD group under the biomarker scenario. Therefore, spending on people with diabetes falls. We estimate the highest costs among those with diabetes and stage 5 CKD ($127,730 at baseline).

**Fig 4:**
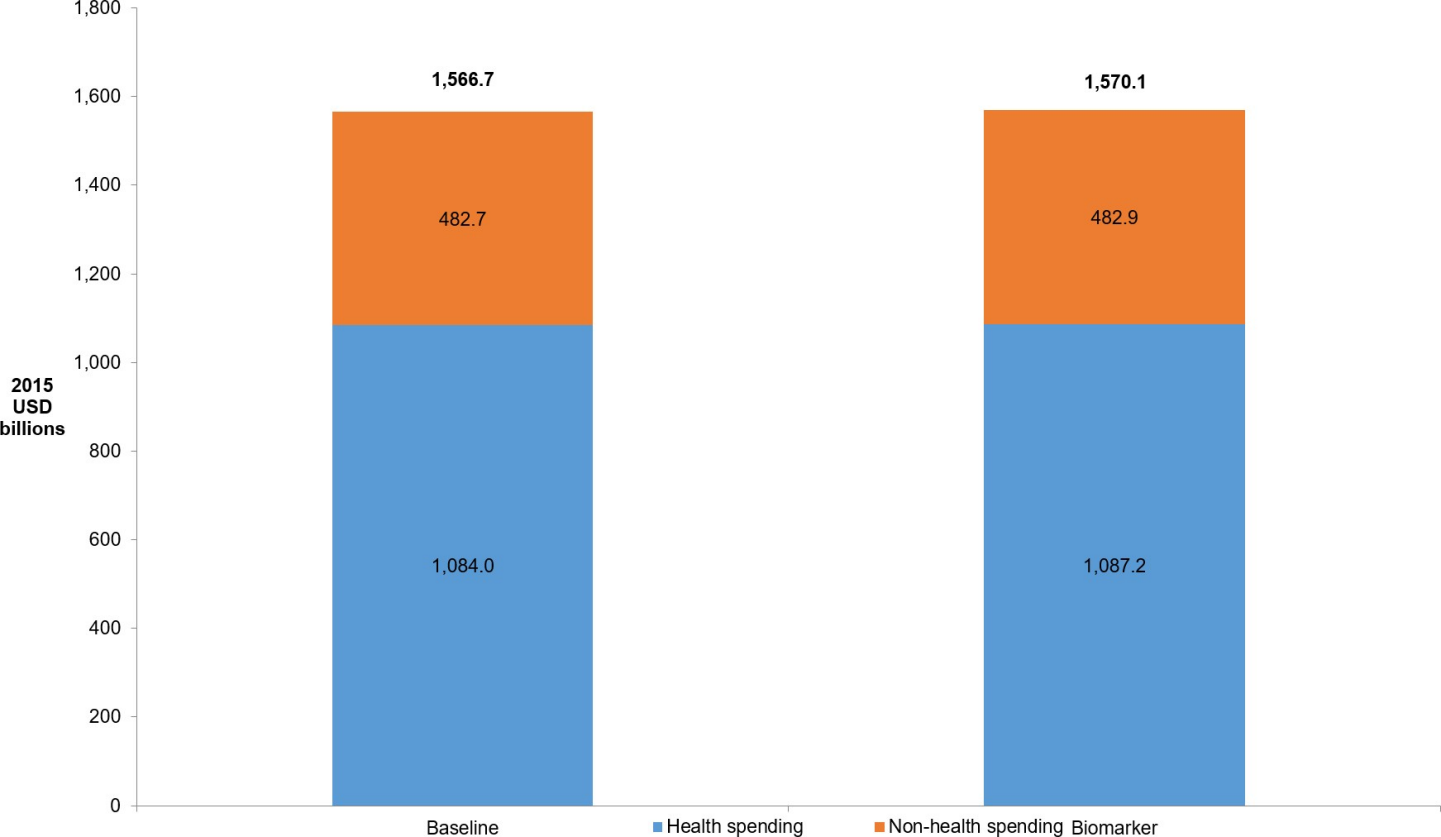
Total annual government health versus non-health spending on people with diabetes. Notes: Reported annual healthcare expenditures are the average values across all years of the simulation (2010–2050). DKD indicates diabetic kidney disease; CKD, chronic kidney disease.

Overall, we estimate total government spending on people with diabetes would rise by 0.2% with the implementation of the biomarker, from $1,566.7 to $1,570.1 billion (Fig 4). This increase stems from a 0.3% increase in healthcare spending, from $ 1,084.0 to $1,087.2 billion, and a 0.04% increase in non-healthcare spending, from $482.7 to $482.9 billion. The increases in healthcare and non-healthcare spending both stem from longer life expectancy in the biomarker scenario, which gives people with diabetes additional time to collect pensions and incur healthcare costs. Calculating the cost-effectiveness, we find that the implementation of the biomarker would cost $12,796 per life year and $25,842 per QALY.

The results of the scenario analyses in which we examined how the biomarker’s effects would change when combined with more effective treatment are presented in Table 1. Life expectancy gains would be larger– 0.2 years with the biomarker alone, 0.3 years when combined with 50% more effective treatment, and 0.4 years when combined with 100% more effective treatment. Given the more effective treatment, the cost savings from preventing and delaying the development of DKD would effectively reduce the increased costs of the biomarker testing in these simulations annual per person healthcare spending on individuals with diabetes fell from $41,067 with the biomarker to $40,902 with the biomarker and 50% more effective treatment, and to $40,739 with the biomarker and 100% more effective treatment. Similarly, total government healthcare spending on individuals with diabetes would be reduced: from $1.570 trillion with the biomarker to $1.568 trillion with the addition of 50% more effective treatment and $1.565 trillion with 100% more effective treatment. Of course the ultimate effects of more effective treatment on healthcare costs would depend on the treatment’s incremental cost over currently available treatments.

**Table 1:** Results of additional scenario analyses.

	Scenario			
Outcome	Baseline	Biomarker	Biomarker + 50% better treatment	Biomarker + 100% better treatment
**Prevalence of DKD (baseline = 100)**	100.00	95.17	92.61	90.00
**Prevalence of stage 5 CKD among people with diabetes (baseline = 100)**	100.00	97.03	95.15	93.77
**Life expectancy from age 51 of people with diabetes**	30.5	30.7	30.8	30.9
**Expected QALYs from age 51 of people with diabetes**	18.8	18.9	18.9	19.0
**Per person medical spending on people with diabetes**	41,179	41,067	40,902	40,739
**Total government health spending on people with diabetes (2015 USD billions)**	1,084	1,087	1,085	1,082
**Total government non-health spending on people with diabetes (2015 USD billions)**	482.7	482.9	483.0	483.0
**Total government spending on people with diabetes (2015 USD billions)**	1,567	1,570	1,568	1,565

Note: Outcomes represent the average across all years of the simulation (2010–2050). DKD indicates diabetic kidney disease; CKD, chronic kidney disease.

## Discussion

This study considered the implementation of a novel biomarker test to identify individuals with diabetes at risk of developing DKD and individuals with DKD at risk of rapid progression. Another study found use of personalized and precision medicine innovations to identify at-risk individuals could reduce disease incidence by 10%, generating about $33 to $114 billion in longer, healthier lives in the US.[40] Although that study considered a broad set of innovations while ours is narrower, it is notable that both generated similar predicted reductions in disease incidence. Specifically, according to our simulations, the biomarker test could reduce the prevalence of DKD by 6% by 2050 and the prevalence of diabetes with stage 5 CKD by 8% below current projections using present-day standard of care. Averaging over the study cohorts from 2010 to 2050, this would translate to a gain of 0.2 years of life expectancy and 0.1 additional expected QALYs among individuals with diabetes. Our results are similar to those of Critselis et al. 2018, who found that screening patients with diabetes based on the CKD273 classifier would result in 0.13 QALYs gained.[41] Further, we estimate per person annual healthcare costs would be expected to decline by 0.3% due to the delayed and prevented progression to DKD and stage 5 CKD, which impose higher per person costs than diabetes without DKD. Accounting for the total changes in government healthcare, disability, and pension spending, we estimate government spending on people with diabetes could be expected to rise by less than one percent, chiefly due to the increased life expectancy.

Although effective treatments currently exist for DKD, outcomes often remain poor because patients may go undiagnosed and untreated until serious complications manifest.[11–13] Therefore, there is an unmet need for earlier diagnosis and treatment which an effective biomarker test could help fill. Novel prognostic biomarkers, such as sTNFR1, may increase the ability of providers to identify patients that are at high risk of developing DKD or, for patients already diagnosed with DKD, at high risk of rapidly progressing.[15, 16] This improved detection would directly address some of the unmet need among patients with diabetes.

Our study results indicate that the implementation of a novel biomarker test to identify at-risk patients sooner could present a cost-effective way to slow the development and progression of DKD, thereby improving patient outcomes. In particular, we estimate the implementation of biomarker testing in the diabetes population would cost $12,796 per life year gained and $25,842 per QALY gained, well below commonly used cost effectiveness thresholds in the US.[42] Much of this benefit derives from increasing use of currently available treatments. Our scenario analyses demonstrate the potential health gains would be larger when combined with more effective treatment, although the value such a treatment presents would of course depend on its efficacy and cost.

Recently, the Canagliflozin and Renal Endpoints in Diabetes with Established Nephropathy Clinical Evaluation (CREDENCE) trial demonstrated a lower risk of kidney failure in patients with type 2 diabetes and chronic kidney disease with the sodium glucose co-transporter 2 (SGLT2) inhibitor canagliflozin versus placebo.[43] The trial found a 30% lower risk of the primary composite outcome, which consisted of end-stage renal disease (dialysis, transplantation, or a sustained estimated glomerular filtration rate of <15 mL/min/1.73 m2), doubling of serum creatinine, or renal or cardiovascular death, when treated with canagliflozin versus placebo.[43] Notably, all patients were required to receive optimized standard of care with an angiotensin-converting-enzyme inhibitor or angiotensin II receptor blocker, suggesting benefit beyond that provided by current treatments. Data from large cardiovascular outcomes trials have suggested a potential benefit of treatment with SGLT2 inhibitors on renal outcomes, but these trials generally enrolled patients with relatively healthy kidney function and accrued few hard renal outcomes.[44–46] Ongoing studies with other SGLT2 inhibitors to evaluate potential benefits on chronic kidney disease in patients with and without type 2 diabetes will be needed to confirm whether these results represent a class effect [47, 48], and further analyses would be needed to forecast the long-term effects on progression of DKD.

Strengths of this study include its foundation in the validated THEMIS model [24, 49–51], which allows for the simulation of a variety of policy-relevant health and economic outcomes based on a real-world population. This modeling environment is ideal for studying changes during an individual’s life cycle and understanding their long-term implications for health and government spending. In addition, given the growing prevalence of diabetes and DKD,[5, 52] this study addresses an important and timely question.

The study also has limitations. For example, the THEMIS model assumes that health conditions follow a Markovian process, so that future health, mortality, and functional outcomes are entirely determined by the risk factors and conditions in the prior period. While this method has been demonstrated to fit well for simulations during the 1998 to 2014 period, we cannot be certain the fit would remain high over the longer period. THEMIS also uses a variety of macro-economic projections from various bodies (SSA, CBO, Census, etc.). Since both baseline and intervention scenarios use the same projections, any errors in these projections will impact all the results in the same way and thus can be accounted for by focusing the results on relative differences between the intervention and baseline scenarios.

Beyond the general limitations of THEMIS, the construction of the DKD module for THEMIS also has limitations. Although the use of real-world data as the foundation of the simulation allows us to capture many of the heterogeneous characteristics of the represented population, we were not able to capture all different stages among the DKD population. The HRS data which serves as the basis for THEMIS lacks clinical detail, such as laboratory values, which necessitated estimating DKD status in NHANES and using this estimation to impute DKD status in THEMIS. Because a limited set of demographic and health variables were available for this imputation, we were only able to model DKD as non-stage 5 CKD versus stage 5 CKD. Therefore, this study understates the negative consequences of progression between stages 1–4 and its results are conservative, as costs start to increase at earlier stages.[53] Future work should explore the potential for earlier diagnosis and treatment to reduce progression within the 5 stages of DKD.

In addition, the relatively small sample of patients in NHANES compared to large administrative datasets limits the precision of estimated prevalence. Furthermore, the NHANES survey only includes a single measure of serum creatinine and urine albumin. The Kidney Disease Improving Global Outcomes work group guidelines state that two abnormal measures over at least 90 days are necessary to definitively determine CKD. Therefore, it is possible that our method produced some misclassification. In addition, its cross-sectional nature imposed challenges for us in modeling the incidence of DKD (with and without stage 5 CKD), for which panel data would be preferable. We addressed this limitation by using incidence rates from longitudinal claims data and matching prevalence rates to NHANES estimates. Despite these limitations, NHANES was the best available data source for this imputation at the present time. With its nationally representative population and available laboratory data, NHANES is widely used for measuring the prevalence of undiagnosed disease in the US.[54, 55]

Based on the findings of this study, the implementation of a novel biomarker to identify individuals at risk of developing DKD or progressing rapidly could allow for earlier diagnosis and intervention, thereby reducing the prevalence of DKD (with and without stage 5 CKD) and increasing life expectancy among people with diabetes (with or without DKD). In summary, use of the biomarker combined with more effective treatment or increased use of current treatments offers the possibility for a longer, healthier life for people with diabetes, while raising healthcare spending by less than one percent.

## Supporting information

S1 Appendix The health economics medical innovation simulation: Technical documentation. (PDF)

S2 Appendix Implementation of THEMIS for this study. (DOCX)

S3 Appendix Marginal effects of demographic factors. (XLSX)
