# Supplementary material for: Lifetime benefits of early detection and treatment of diabetic kidney disease
Source: PLoS One. 2019 May 31;14(5):e0217487. doi: 10.1371/journal.pone.0217487 (PMC6544227; doi:10.1371/journal.pone.0217487)
Supplement: S1 Appendix — (PDF) [file pone.0217487.s001.pdf]

The Health Economics Medical Innovation Simulation: Technical Documentation

Contents

1 Functioning of the dynamic model **6**
1.1 Background . . . 6
1.2 Overview . . . 7
1.3 Comparison with other prominent microsimulation models of health expenditures . . . 8
1.3.1 Congressional Budget Office Long-Term Model . . . 8
1.3.2 Centers for Medicare & Medicaid Services . . . 9

2 Data sources used for estimation **9**
2.1 Health and Retirement Study . . . 10
2.2 National Health Interview Survey . . . 10
2.3 Medical Expenditure Panel Survey . . . 11
2.4 Medicare Current Beneficiary Survey . . . 11

3 Data sources for trends and baseline scenario **12**
3.1 Data for trends in entering cohorts . . . 12
3.2 Data for other projections . . . 12
3.3 Demographic adjustments . . . 12

4 Estimation **13**
4.1 Transition model . . . 13
4.1.1 Inverse hyperbolic sine transformation . . . 14
4.2 Quality-adjusted life years . . . 15

5 Model for new cohorts **15**
5.1 Information available and empirical strategy . . . 16
5.2 Model and estimation . . . 16

6 Government revenues and expenditures **17**
6.1 Old-Age and Survivors Insurance benefits . . . 17
6.2 Social Security Disability Insurance benefits . . . 18
6.3 Supplemental Security Income benefits . . . 18
6.4 Medical costs estimation . . . 18
6.5 Taxes . . . 19

7 Scenarios and robustness **20**
7.1 Obesity reduction scenario . . . 20

8 Implementation **20**
8.1 Intervention module . . . 21

9 Model development **21**
9.1 Transition model . . . 22
9.2 Quality-adjusted life years . . . 23
9.2.1 Health-related quality-of-life . . . 23
9.2.2 HRQoL in the MEPS . . . 23
9.2.3 MEPS-HRS crosswalk development . . . 23
9.3 Drug expenditures . . . 24

9.3.1 Drug expenditures - MEPS . . . 24
9.3.2 Drug expenditures - MCBS . . . 25
9.3.3 Drug expenditures - estimation . . . 25

10 Validation **25**
10.1 Cross-validation . . . 26
10.2 External validation . . . 26
10.2.1 Benefits from Social Security Administration . . . 26
10.2.2 Benefits from Medicare and Medicaid . . . 26
10.3 External corroboration . . . 27

11 Baseline forecasts **27**
11.1 Disease prevalence . . . 27

12 Tables **30**

References **52**

List of figures

A Architecture of THEMIS . . . 9
B Distribution of EQ-5D index scores for ages 51+ in the 2001 MEPS . . . 24
C Historic and forecasted chronic disease prevalence for men 55+ . . . 28
D Historic and forecasted chronic disease prevalence for women 55+ . . . 28
E Historic and forecasted ADL and IADL prevalence for men 55+ . . . 29
F Historic and forecasted ADL and IADL prevalence for women 55+ . . . 29

List of tables

A Health condition prevalences in survey data . . . 31
B Survey questions used to determine health conditions . . . 32
C Data sources and methods for projecting future cohort trends . . . 33
D Projected baseline trends for future cohorts . . . 34
E Prevalence of obesity, hypertension, diabetes and current smokers among ages 46-56 in 1978 and 2004 . . . 35
F Outcomes in the transition model . . . 36
G Restrictions on transition model . . . 37
H Desciptive statistics for exogeneous control variables . . . 38
I Crossvalidation of 1998 cohort: Simulated vs reported mortality and nursing home outcomes in 2000, 2006, and 2012 . . . 39
J Crossvalidation of 1998 cohort: Simulated vs reported demographic outcomes in 2000, 2006, and 2012 . . . 39
K Crossvalidation of 1998 cohort: Simulated vs reported binary health outcomes in 2000, 2006, and 2012 . . . 39
L Crossvalidation of 1998 cohort: Simulated vs reported risk factor outcomes in 2000, 2006, and 2012 . . . 40
M Crossvalidation of 1998 cohort: Simulated vs reported binary economic outcomes in 2000, 2006, and 2012 . . . 40
N Crossvalidation of 1998 cohort: Simulated vs reported continuous economic outcomes in 2000, 2006, and 2012 . . . 40
O Prevalence of Instrumental Activities of Daily Living (IADL) and Activities of Daily Living (ADL) limitations among ages 51+ in the Medical Expenditure Panel Survey (MEPS) 2001 and HRS 1998 . . . 41
P Prevalence of IADL limitations and physical function limitations among ages 51+ in MEPS 2001 and HRS 1998 . . . 41
Q OLS regressions of EQ-5D utility index among ages 51+ in the MEPS 2001 . . . 42
R OLS regression of the predicted EQ-5D index score against chronic conditions and THEMIS-type functional status specification . . . 43
S Average predicted EQ-5D score, age, and prevalence of chronic conditions by functional status for the stock THEMIS simulation sample . . . 44
T Initial conditions used for estimation (1992) and simulation (2010) . . . 45
U Parameter estimates for latent model: conditional means and thresholds . . . 46
V Parameter estimates for latent model: parameterized covariance matrix . . . 47
W Per capita medical spending by payment source, age group, and year . . . 48
X Simulation results for status quo scenario . . . 48

Y Simulation results for obesity reduction scenario compared to status quo 49
Z Assumptions for each calendar year 50
AA Assumptions for each birth year 51

Acronyms

ndix

ACA Affordable Care Act

ADL Activities of Daily Living

AIME Average Indexed Monthly Earnings

BMI Body Mass Index

CBO Congressional Budget Office

CMS Centers for Medicare & Medicaid Services

COLA Cost of Living Adjustment

CPI Consumer Price Index

EQ-5D EuroQol Five Dimensions Questionnaire

FEM Future Elderly Model

GDP Gross Domestic Product

HRQoL Health-Related Quality of Life

HRS Health and Retirement Study

IADL Instrumental Activities of Daily Living

MCBS Medicare Current Beneficiary Survey

MEPS Medical Expenditure Panel Survey

MEPS-HC Medical Expenditure Panel Survey Household Component

NHEA National Health Expenditure Accounts

NHIS National Health Interview Survey

NRA Normal Retirement Age

OASI Old-Age and Survivors Insurance

OECD Organization for Economic Co-operation and Development

OLS Ordinal Least Squares

OOP Out-of-Pocket

PIA Primary Insurance Amount

QALY Quality-Adjusted Life Year

SF-12 12-Item Short Form Health Survey

SF-6D Short-Form 6D

SGA Substantial Gainful Activity

SSA Social Security Administration

SSDI Social Security Disability Insurance

SSI Supplemental Security Income

THEMIS The Health Economics Medical Innovation Simulation

UK United Kingdom

US United States

1 Functioning of the dynamic model

1.1 Background

The Health Economics Medical Innovation Simulation (THEMIS) is a microsimulation model originally developed out of an effort to examine health and health care costs among the elderly Medicare population (age 65+). A description of the previous incarnation of the model, called the Future Elderly Model (FEM), can be found in Goldman et al. (12). The original work was founded by the Centers for Medicare & Medicaid Services (CMS) and carried out by a team of researchers composed of Dana P. Goldman, Paul G. Shekelle, Jayanta Bhattacharya, Michael Hurd, Geoffrey F. Joyce, Darius N. Lakdawalla, Dawn H. Matsui, Sydne J. Newberry, Constantijn W. A. Panis and Baoping Shang.

Since then various extensions have been implemented to the original model. The most recent version now projects health outcomes for all Americans aged 51 and older and uses the Health and Retirement Study (HRS) as a host dataset rather than the Medicare Current Beneficiary Survey (MCBS). The work has also been extended to include economic outcomes such as earnings, labor force participation and pensions. This work was funded by the National Institute on Aging through its support of the RAND Roybal Center for Health Policy Simulation (P30AG024968), the Department of Labor through contract J-9-P-2-0033, the National Institutes of Aging through the R01 grant "Integrated Retirement Modeling" (R01AG030824) and the MacArthur Foundation Research Network on an Aging Society. Finally, the computer code of the model was transferred from Stata to C++. This report incorporates these new development efforts in the description of the model.

Technical documentation for the RAND FEM can be found in Goldman et al. (12). THEMIS has been developed and derived from an earlier version of the RAND FEM.

1.2 Overview

THEMIS was developed through partnership with government agencies to assess important policy questions. The initial funding came from the CMS to develop a model that would assist the trustees of Medicare in analyzing the impact of new medical technologies on the future health, longevity, and health spending of Medicare beneficiaries in the United States (US). The output of the CMS project was a special issue of Health Affairs (published on September 26, 2005), devoted exclusively to the model and its findings. Additional funding from the National Institutes of Health and the US Department of Labor has been used to expand the model to develop additional policy applications.

The model has an extensive record of use by government agencies, Advisory Committees and policymakers to inform decisions. The Congressional Budget Office (CBO) has found the output to be a valuable resource when considering microsimulations of the US population, economy, and federal budget. The CBO also has relied on the model for simulations of various health trends (obesity and smoking). The Committee on National Statistics of the National Research Council highlighted the model in a 2010 publication as the only example of a microsimulation model that can produce health care cost projections, being the largest and most commonly used microsimulation model in the literature (5). In one influential study, the model was used to estimate the value of a complete cessation of smoking among Medicare beneficiaries (10). The results of the model demonstrated that this would increase Medicare program spending slightly due to increases in life expectancy that outweigh tobacco-related health spending reductions; these results were used by the CBO in considering the effects of potentially raising excise taxes on cigarettes (22). The National Committee on Vital and Health Statistics of the Department of Health and Human Services Subcommittee on Population Health highlighted how the use of real-world longitudinal data is a key advantage of the model in predicting how individuals transition from one health state to another (6). In testimony before the US Senate Committee on Health Education, results from the model on spending associated with obesity were used to argue for further efforts to reduce obesity in order to slow the rise in health care spending (33).

In addition, the model has an extensive record of publications in high-impact peer-reviewed journals. A study published in the American Journal of Public Health in 2009 used the model to analyze the economic impact of several prevention scenarios for obesity, smoking, diabetes, and hypertension, finding that effective prevention could substantially improve the health of Americans, with little or no additional lifetime medical spending (13). Several studies using the model to examine policies to reduce obesity have been published in high-impact peer-reviewed journals including two studies in Health Affairs and one study in the Journal of Health Economics on the value of specific medical and pharmaceutical interventions to reduce obesity (9; 16; 21). Additional work on the fiscal implications of smoking and obesity has been published in the National Tax Journal (11), and the Forum for Health Economics and Policy (10). A study published in Health Affairs in 2009 examining the effects of different pharmaceutical policies on innovation using the model was awarded the annual Garfield Economic Impact Award for outstanding research that demonstrates how health research impacts the economy (15). The model has also been used in cancer prevention studies: an article published in Health Affairs looked at the economic implications of cancer prevention, drawing conclusions for the financial health of the Medicare program (2). Another article published in Health Affairs by a Centers for Disease Control and Prevention author, used results from the model to conclude that neither technological advances nor improved functional status among the elderly would be likely to relieve budgetary pressures on the Medicare program (18).

The defining characteristic of the model is the modeling of real rather than synthetic cohorts, all of whom are followed at the individual level. This allows for more heterogeneity in behavior

than would be allowed by a cell-based approach. Also, since the HRS interviews both respondent and spouse, we can link records to calculate household-level outcomes such as net income and Social Security retirement benefits, which depend on the outcomes of both spouses. The omission of the population younger than age 51 sacrifices little generality, since the bulk of expenditure on the public programs we consider occurs after age 50. However, we may fail to capture behavioral responses among the young.

The model has three core components:

- The initial cohort module predicts the economic and health outcomes of new cohorts of 51/52 year-olds. This module takes in data from the HRS and trends calculated from other sources. It allows us to "generate" cohorts as the simulation proceeds, so that we can measure outcomes for the age 51+ population in any given year.

- The transition module calculates the probabilities of transiting across various health states and financial outcomes. The module takes as inputs risk factors such as smoking, weight, age and education, along with lagged health and financial states. This allows for a great deal of heterogeneity and fairly general feedback effects. The transition probabilities are estimated from the longitudinal data in the HRS.

- The policy outcomes module aggregates projections of individual-level outcomes into policy outcomes such as taxes, medical care costs, pension benefits paid, and disability benefits. This component takes account of public and private program rules to the extent allowed by the available outcomes. Because we have access to data on employer pension plans in the HRS, we are able to realistically model retirement benefit receipt.

Figure A provides a schematic overview of the model. We start in 2004 with an initial population aged 51+ taken from the HRS. We then predict outcomes using our estimated transition probabilities (see section 4.1). Those who survive make it to the end of that year, at which point we calculate policy outcomes for the year. We then move to the following time period (two years later), when a new cohort of 51 and 52 year-olds enters (see section 5.1). This entrance forms the new age 51+ population, which then proceeds through the transition model as before. This process is repeated until we reach the final year of the simulation.

1.3 Comparison with other prominent microsimulation models of health expenditures

THEMIS is unique among existing models that make health expenditure projections. It is the only model that projects health trends rather than health expenditures. It is also the only model that generates mortality out of assumptions on health trends rather than historical time series.

1.3.1 Congressional Budget Office Long-Term Model

The CBO uses time-series techniques to project health expenditure growth in the short term and then makes an assumption on long term growth. They use a long term growth of excess costs of 2.3 percentage points starting in 2020 for Medicare. They then assume a reduction in excess cost growth in Medicare of 1.5% through 2083, leaving a rate of 0.9% in 2083. For non-Medicare spending they assume an annual decline of 4.5%, leading to an excess growth rate in 2083 of 0.1%.

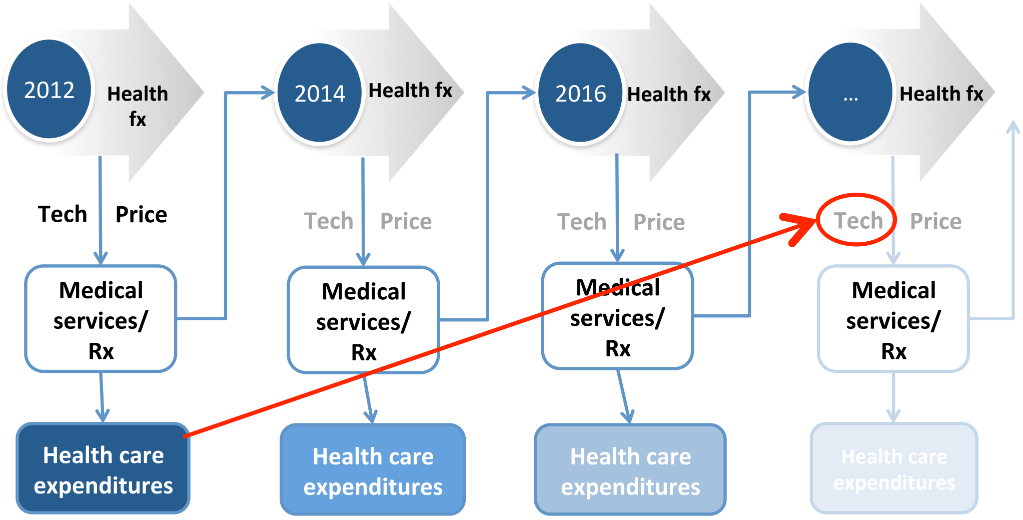

Figure A: Architecture of THEMIS

1.3.2 Centers for Medicare & Medicaid Services

CMS performs an extrapolation of medical expenditures over the first ten years, then computes a general equilibrium model for years 25 through 75 and linearly interpolates to identify medical expenditures in years 11 through 24 of their estimation. The core assumption they use is that excess growth of health expenditures will be one percentage point higher per year for years 25-75 (that is if nominal Gross Domestic Product (GDP) growth is 4%, health care expenditure growth will be 5%).

2 Data sources used for estimation

The HRS is the main data source for the model. We supplemented this data with health trends and health care costs coming from 3 major health surveys in the U.S. We describe these surveys below and the samples we selected for the analysis. We first list the variables used in the analysis. We then give details on the data sources.

Estimated Outcomes in Initial Conditions Model

**Economic Outcomes**	**Health Outcomes**
Employment	Hypertension
Earnings	Heart Disease
Wealth	Self-Reported Health
Defined Contribution Pension Wealth	Body Mass Index Status
Pension Plan Type	Smoking Status
Averaged Indexed Monthly Earnings	Functional Status
Social Security Quarters of Coverage	
Health Insurance	

Estimated Outcomes in/from Transition Model

**Economic Outcomes**	**Health Outcomes**	**Other Outcomes**
Employment	Death	Income Tax Revenue
Earnings	Heart	Social Security Revenue
Wealth	Stroke	Medicare Revenue
Demographics	Cancer	Medical Expenses
Health Insurance	Hypertension	Medicare Part A Expenses
SSDI Claim	Diabetes	Medicare Part B Expenses
Defined Benefit Claim	Lung Disease	Medicare Part B Enrollment
SSI Claim	Nursing Home	Medicare Part D Enrollment
OASI Claim	BMI	OASI Enrollment
	Smoking Status	SSDI Enrollment
	ADL Limitations	SSI Enrollment
	IADL Limitations	Medicaid Enrollment
		Medicaid Expenditures

2.1 Health and Retirement Study

The HRS waves 2000-2012 are used to estimate the transition model. Interviews occur every two years. We use the dataset created by RAND (RAND HRS, version O) as our basis for the analysis. We use all cohorts in the analysis and consider sampling weights whenever appropriate. When appropriately weighted, the HRS in 2010 is representative of U.S. households where at least one member is at least 51. The HRS is also used as the host data for the simulation (pop 51+ in 2010) and for new cohorts (aged 51 and 52 in 2010).

The HRS adds new cohorts every six years. The latest available cohort was added in 2010, which is why that is THEMIS's base year.

2.2 National Health Interview Survey

The National Health Interview Survey (NHIS) contains individual-level data on height, weight, smoking status, self-reported chronic conditions, income, education, and demographic variables. It is a repeated cross-section done every year for several decades. But the survey design has been

significantly modified several times. Before year 1997, different subgroups of individuals were asked about different sets of chronic conditions, after year 1997, a selected sub-sample of the adults were asked a complete set of chronic conditions. The survey questions are quite similar to that in the HRS. As a result, for projecting the trends of chronic conditions for future 51/52 year-olds, we only use data from 1997 to 2010. A review of survey questions is provided in Table B Information on weight and height were asked every year, while information on smoking was asked in selected years before year 1997, and has been asked annually since year 1997.

THEMIS uses the NHIS to project prevalence of chronic conditions in future cohorts of 51/52 year olds. The method is discussed in Sections 3.1 and 5.1. THEMIS also relies on the MEPS, a subsample of NHIS respondents, for model estimation. See section 2.3 for a description.

2.3 Medical Expenditure Panel Survey

The MEPS, beginning in 1996, is a set of large-scale surveys of families and individuals, their medical providers (doctors, hospitals, pharmacies, etc.), and employers across the US The Medical Expenditure Panel Survey Household Component (MEPS-HC) provides data from individual households and their members, which is supplemented by data from their medical providers. The MEPS-HC collects data from a representative sub sample of households drawn from the previous year's NHIS. Since the NHIS does not include the institutionalized population, neither does the MEPS: this implies that we can only use the MEPS to estimate medical costs for the non-elderly population. Information collected during household interviews include: demographic characteristics, health conditions, health status, use of medical services, sources of medical payments, and body weight and height. Each year the household survey includes approximately 12,000 households or 34,000 individuals. Sample size for those aged 51-64 is about 4,500. MEPS has comparable measures of Social Economic Status variables as those in the HRS, including age, race/ethnicity, educational level, census region, and marital status.

THEMIS uses the MEPS years 2000-2012 for cost estimation. See Section 6.4 for a description. THEMIS also uses the MEPS 2001 data for Quality-Adjusted Life Year (QALY) model estimation. This is described in Section 4.2.

2.4 Medicare Current Beneficiary Survey

The MCBS is a nationally representative sample of aged, disabled and institutionalized Medicare beneficiaries. The MCBS attempts to interview each respondent twelve times over three years, regardless of whether he or she resides in the community, a facility, or transitions between community and facility settings. The disabled (under 65 years of age) and oldest-old (85 years of age or older) are over-sampled. The first round of interviewing was conducted in 1991. Originally, the survey was a longitudinal sample with periodic supplements and indefinite periods of participation. In 1994, the MCBS switched to a rotating panel design with limited periods of participation. Each fall a new panel is introduced, with a target sample size of 12,000 respondents and each summer a panel is retired. Institutionalized respondents are interviewed by proxy. The MCBS contains comprehensive self-reported information on the health status, health care use and expenditures, health insurance coverage, and socioeconomic and demographic characteristics of the entire spectrum of Medicare beneficiaries. Medicare claims data for beneficiaries enrolled in fee-for-service plans are also used to provide more accurate information on health care use and expenditures. MCBS years 1992-2012 are used for estimating medical cost and enrollment models. See section 6.4 for discussion.

3 Data sources for trends and baseline scenario

Two types of trends need to be projected in the model. First, we need to project trends in the incoming cohorts (the future new age 51/52 individuals). This includes trends in health and economic outcomes. Second, we need to project excess aggregate growth in real income and excess growth in health spending.

3.1 Data for trends in entering cohorts

We use a multitude of data sources to compute US trends. First, we use the NHIS for chronic conditions and apply the methodology discussed in Goldman et al. (12). The method consists of projecting the experience of younger cohorts into the future until they reach age 51. The projection method is tailored to the synthetic cohorts observed in the NHIS. For example, in 1980 we observe a representative sample of age 35 individuals born in 1945. We follow their disease patterns from 1980 to 1981 by then selecting from the survey those aged 36 in 1981, accounting for mortality, etc.

We then collect information on other trends, i.e., for obesity and smoking, from other studies (14; 17; 20; 25; 27). Table C presents the sources and Table D presents the trends we use in the baseline scenario. Table E presents the prevalence of obesity, hypertension, diabetes, and current smokers in 1978 and 2004, and the annual rates of change from 1978 to 2004. We refer the readers to the analysis in Goldman et al. (12) for information on how the trends were constructed.

3.2 Data for other projections

We make two assumptions relating to real growth in wages and medical costs. Firstly, as is done in the 2009 Social Security Trustees report (30) intermediate cost scenario, we assume a long term real increase in wages (earnings) of 1.1% per year. Next, following CMS, we assume excess real growth in medical costs (that is additional cost growth to GDP growth) as 1.5% in 2004, reducing linearly to 1% in 2033, .4% in 2053, and -.2% in 2083. We also include the Affordable Care Act (ACA) cost growth targets as an optional cap on medical cost growth. Baseline medical spending figures presented assume those targets are met. GDP growth in the near term (through 2019) is based on CBO projections, with the OASDI Trustees assumption of 2% yearly afterwards (30).

3.3 Demographic adjustments

We make two adjustments to the weighting in the HRS to match population counts. Since we deleted some cases from the data due to missing responses and other data quality concerns, this accounts for selectivity based on these characteristics. First, we post-stratify the HRS sample by 5 year age groups, gender and race and rebalance weights using the Census Bureau 2000-2010 Intercensal Population Estimates. We do this for both the host data set and the new cohorts. We scale the weights for future new cohorts using 2012 National Population Projections based on race and gender (32). Second, we post- stratify the HRS sample of deaths between the 2002 and 2004 interview waves by 5 year age groups, gender and race and rebalance weights based on the Human Mortality Database.

Once the simulation begins, trends in migration and mortality are applied. We use net migration from the Social Security Administration (SSA) Trustees report intermediate cost scenario (30). Seperate mortality rate adjustment factors are defined for the under and over 65 age groups based on the mortality projections from the 2013 SSA Trustees report. The SSA projections are interpolated through 2090, then extended using generalized least squares regression with log link through 2150.

4 Estimation

In this section we describe the approach used to estimate the transition model, the core of the THEMIS, and the initial cohort model which is used to rejuvenate the simulation population.

4.1 Transition model

We consider a large set of outcomes for which we model transitions. Table F gives the set of outcomes considered for the transition model along with descriptive statistics and the population at risk when estimating the relationships.

Since we have a stock sample from the age 51+ population, each respondent goes through an individual-specific series of intervals. Hence, we have an unbalanced panel over the age range starting from 51 years old. Denote by j_{i0} the first age at which respondent i is observed and j_{iT_i} the last age when he is observed. Hence we observe outcomes at ages $j_i = j_{i0}, \ldots, j_{iT_i}$.

We first start with discrete outcomes which are absorbing states (e.g., disease diagnostic, mortality, benefit claiming). Record as $h_{i,j_i,m} = 1$ if the individual outcome m has occurred as of age j_i. We assume the individual-specific component of the hazard can be decomposed in a time invariant and variant part. The time invariant part is composed of the effect of observed characteristics x_i that are constant over the entire life course and initial conditions $h_{i,j_0,-m}$ (outcomes other than the outcome m) that are determined before the first age in which each individual is observed.[1] The time-varying part is the effect of previously diagnosed outcomes $h_{i,j_i-1,-m}$ on the hazard for m.[2] We assume an index of the form $z_{m,j_i} = x_i\beta_m + h_{i,j_i-1,-m}\gamma_m + h_{i,j_0,-m}\psi_m$. Hence, the latent component of the hazard is modeled as

$$h^*_{i,j_i,m} = x_i\beta_m + h_{i,j_i-1,-m}\gamma_m + h_{i,j_0,-m}\psi_m + a_{m,j_i} + \varepsilon_{i,j_i,m} \quad (1)$$

$$m = 1, \ldots, M_0,\ j_i = j_{i0}, \ldots, j_{i,T_i},\ i = 1, \ldots, N$$

The term $\varepsilon_{i,j_i,m}$ is a time-varying shock specific to age j_i. We assume that this last shock is normally distributed and uncorrelated across diseases. We approximate a_{m,j_i} with an age spline. After several specification checks, knots at age 65 and 75 appear to provide the best fit. This simplification is made for computational reasons since the joint estimation with unrestricted age fixed effects for each condition would imply a large number of parameters. The absorbing outcome, conditional on being at risk, is defined as

$$h_{i,j_i,m} = \max\{I(h^*_{i,j_i,m} > 0), h_{i,j_i-1,m}\}$$

the occurrence of mortality censors observation of other outcomes in a current year. Mortality is recorded from exit interviews.

A number of restrictions are placed on the way feedback is allowed in the model. Table G documents restrictions placed on the transition model. We also include a set of other controls. A list of such controls is given in Table H along with descriptive statistics.

We have three other types of outcomes:

1. First, we have binary outcomes which are not an absorbing state, such as living in a nursing home. We specify latent indices as in Equation (1) for these outcomes as well but where the lag dependent outcome also appears as a right-hand side variable. This allows for state-dependence.

[1]Section 9.1 explains why the $h_{i,j_0,-m}$ terms are included.

[2]With some abuse of notation, $j_i - 1$ denotes the previous age at which the respondent was observed.

2. Second, we have ordered outcomes. These outcomes are also modeled as in Equation (1) recognizing the observation rule is a function of unknown thresholds ς_m. Similarly to binary outcomes, we allow for state-dependence by including the lagged outcome on the right-hand side.

3. The third type of outcomes we consider are censored outcomes, earnings and financial wealth. Earnings are only observed when individuals work. For wealth, there are a non-negligible number of observations with zero and negative wealth. For these, we consider two part models where the latent variable is specified as in Equation (1) but model probabilities only when censoring does not occur. In total, we have M outcomes.

The parameters $\theta_1 = \left(\{\beta_m, \gamma_m, \psi_m, \varsigma_m\}_{m=1}^{M}\right)$ can be estimated by maximum likelihood. Given the normality distribution assumption on the time-varying unobservable, the joint probability of all time-intervals until failure, right-censoring or death conditional on the initial conditions $h_{i,j_0,-m}$ is the product of normal univariate probabilities. Since these sequences, conditional on initial conditions, are also independent across diseases, the joint probability over all disease-specific sequences is simply the product of those probabilities.

For a given respondent observed from initial age j_{i0} to a last age j_{T_i}, the probability of the observed health history is (omitting the conditioning on covariates for notational simplicity)

$$l_i^{-0}(\theta; h_{i,j_{i0}}) = \left[\prod_{m=1}^{M-1} \prod_{j=j_{i1}}^{j_{T_i}} P_{ij,m}(\theta)^{(1-h_{ij-1,m})(1-h_{ij,M})}\right] \times \left[\prod_{j=j_{i1}}^{j_{T_i}} P_{ij,M}(\theta)\right]$$

We use the -0 superscript to make explicit the conditioning on $\mathbf{h}_{i,j_{i0}} = (h_{i,j_{i0},0}, \ldots, h_{i,j_{i0},M})'$. We have limited information on outcomes prior to this age. The likelihood is a product of M terms with the mth term containing only $(\beta_m, \gamma_m, \psi_m, \varsigma_m)$. This allows the estimation to be done seperately for each outcome.

4.1.1 Inverse hyperbolic sine transformation

One problem fitting the wealth and earnings distribution is that they have a long right tail and wealth has some negative values. We use a generalization of the inverse hyperbolic sine transform presented in MacKinnon and Magee (19). First denote the variable of interest y. The hyperbolic sine transform is

$$y = \sinh(x) = \frac{\exp(x) - \exp(-x)}{2} \tag{2}$$

The inverse of the hyperbolic sine transform is

$$x = \sinh^{-1}(y) = h(y) = \log(y + (1 + y^2)^{1/2})$$

Consider the inverse transformation. We can generalize such transformation, first allowing for a shape parameter θ,

$$r(y) = h(\theta y)/\theta \tag{3}$$

Such that we can specify the regression model as

$$r(y) = x\beta + \varepsilon, \varepsilon \sim \mathrm{N}(0, \sigma^2) \tag{4}$$

A further generalization is to introduce a location parameter ω such that the new transformation becomes

$$g(y) = \frac{h(\theta(y+\omega)) - h(\theta\omega)}{\theta h'(\theta\omega)} \tag{5}$$

where $h'(a) = (1+a^2)^{-1/2}$.

We specify Equation (4) in terms of the transformation g. The shape parameters can be estimated from the concentrated likelihood for θ, ω. We can then retrieve β, σ by standard Ordinal Least Squares (OLS).

Upon estimation, we can simulate

$$\tilde{g} = x\hat{\beta} + \sigma\tilde{\eta}$$

where η is a standard normal draw. Given this draw, we can retransform using Equation (5) and Equation (2)

$$h(\theta(y+\omega)) = \theta h'(\theta\omega)\tilde{g} + h(\theta\omega)$$
$$\tilde{y} = \frac{\sinh\left[\theta h'(\theta\omega)\tilde{g} + h(\theta\omega)\right] - \theta\omega}{\theta}$$

4.2 Quality-adjusted life years

As an alternative measure of life expectancy, we compute a QALY based on the EuroQol Five Dimensions Questionnaire (EQ-5D) instrument, a widely-used Health-Related Quality of Life (HRQoL) measure.[3] The scoring system for EQ-5D was first developed by Dolan (7) using a United Kingdom (UK) sample. Later, a scoring system based on a U.S. sample was generated (29). The HRS does not ask the appropriate questions for computing EQ-5D, but the MEPS does. We use a crosswalk from MEPS to compute EQ-5D scores for HRS respondents not living in a nursing home.[4]

THEMIS has a more limited specification of functional status than what is available in the HRS. In order to predict HRQoL for the THEMIS simulation sample, we needed to build a bridge between THEMIS functional status and the EQ-5D score imputed into HRS. We used OLS to model the EQ-5D score predicted for non-nursing home in the 1998 HRS as a function of the six chronic conditions and the THEMIS specification of functional status. The results are shown in Table R.

The EQ-5D scoring method is based on a community population. Following a suggestion by Emmett Keeler,[5] if a person is living in a nursing home, the QALY is reduced by 10%. We used the parameter estimates in Table R to predict EQ-5D scores for the entire simulation sample and reduced nursing home residents' score by 10%. The resulting scores are representative of the U.S. population (both in community and in nursing homes) aged 51 and over. Table S summarizes the EQ-5D score using this model for the stock simulation sample in 2004.

5 Model for new cohorts

We first discuss the empirical strategy, then present the model and estimation results. The model for new cohorts integrates information coming from trends among younger cohorts with the joint distribution of outcomes in the current population of age 51 respondents in the HRS.

[3]Section 9.2.1 gives some background on HRQoL measures.

[4]Section 9.2.2 describes EQ-5D in the MEPS. Details of the crosswalk model development are given in 9.2.3.

[5]personal correspondance.

5.1 Information available and empirical strategy

For the transition model, we need to first to obtain outcomes listed in Table T. Ideally, we need information on

$$f_t(y_{i1}, \ldots, y_{iM}) = f_t(\mathbf{y}_i)$$

where t denotes calendar time, and $\mathbf{y}_i = (y_{i1}, \ldots, y_{iM})$ is a vector of outcomes of interest whose probability distribution at time t is $f_t()$. Information on how the joint distribution evolves over time is not available. Trends in conditional distributions are rarely reported either.

Generally, we have (from published or unpublished sources) good information on trends for some moments of each outcome (say a mean or a fraction). That is, we have information on $g_{t,m}(y_{im})$, where $g_{t,m}()$ denotes the marginal probability distribution of outcome m at time t.

For example, we know from the NHIS repeated cross-sections that the fraction of individuals that is obese is increasing by roughly 2% a year among 51 year-olds. In statistical jargon this means we have information on how the mean of the marginal distribution of y_{im}, an indicator variable that denotes whether someone is obese, is evolving over time.

We also have information on the joint distribution at one point in time, say year t_0. For example, we can estimate the joint distribution on age 51 respondents in the 1992 wave of the HRS, $f_{t_0}(\mathbf{y}_i)$.

We make the assumption that only some part of $f_t(\mathbf{y}_i)$ evolves over time. In particular, we will model the marginal distribution of each outcome allowing for correlation across these marginals. The correlations will be assumed fixed while the mean of the marginals will be allowed to change over time.

5.2 Model and estimation

Assume the latent model for $\mathbf{y}_i^* = (y_{i1}^*, \ldots, y_{iM}^*)'$

$$\mathbf{y}_i^* = \mu + \varepsilon_i,$$

where ε_i is normally distributed with mean zero and covariance matrix $\mathbf{\Omega}$. It will be useful to write the model as

$$\mathbf{y}_i^* = \mu + \mathbf{L}_\Omega \eta_i$$

where $\mathbf{L}_\Omega$ is a lower triangular matrix such that $\mathbf{L}_\Omega \mathbf{L}_\Omega' = \mathbf{\Omega}$ and $\eta_i = (\eta_{i1}, \ldots, \eta_{iM})'$ are standard normal. We observe $y_i = \Gamma(y_i^*)$ which is a non-invertible mapping for a subset of the M outcomes. For example, we have binary, ordered and censored outcomes for which integration is necessary.

The vector μ can depend on some variables which have a stable distribution over time $\mathbf{z}_i$ (say race, gender and education). This way, estimation preserves the correlation with these outcomes without having to estimate their correlation with other outcomes. Hence, we can write

$$\mu_i = \mathbf{z}_i \beta$$

and the whole analysis is done conditional on $\mathbf{z}_i$.

For binary and ordered outcomes, we fix $\Omega_{m,m} = 1$ which fixes the scale. Also we fix the location of the ordered models by fixing thresholds as $\tau_0 = -\infty$, $\tau_1 = 0$, $\tau_K = +\infty$, where K denotes the number of categories for a particular outcome. We also fix to zero the correlation between selected outcomes (say earnings) and their selection indicator. Hence, we consider two-part models for these outcomes. Because some parameters are naturally bounded, we also re-parameterize the problem

to guarantee an interior solution. In particular, we parameterize

$$\begin{aligned}
\Omega_{m,m} &= \exp(\delta_m),\ m = m_0 - 1, \dots, M \\
\Omega_{m,n} &= \tanh(\xi_{m,n})\sqrt{\Omega_{m,m}\Omega_{m,n}},\ m, n = 1, \dots, N \\
\tau_{m,k} &= \exp(\gamma_{m,k}) + \tau_{k-1},\ k = 2, \dots, K_m - 1, m \text{ ordered}
\end{aligned}$$

and estimate the ($\delta_{m,m}$, $\xi_{m,n}$, γ_k) instead of the original parameters. The parameter values are estimated using the *cmp* package in Stata (26). Table U gives parameter estimates for the indices, while Table V gives parameter estimates of the covariance matrix in the outcomes.

To apply trends to the future cohorts, the latent model is written as

$$\mathbf{y}_i^* = \mu + \mathbf{L}_\Omega \eta_i.$$

Each marginal has a mean change equal to $\mathrm{E}(\mathbf{y} \mid \mu) = (1+\tau)g(\mu)$, where τ is the percent change in the outcome and $g()$ is a non-linear but monotone mapping. Since it is invertible, we can find the vector μ^* where $\mu^* = g^{-1}(\mathrm{E}(y \mid \mu)/(1+\tau))$. We use these new intercepts to simulate new outcomes.

6 Government revenues and expenditures

This gives a limited overview of how revenues and expenditures of the government are computed. These functions are based on 2004 rules, but we include predicted changes in program rules such changes based on year of birth (e.g., Normal Retirement Age (NRA)).

We cover the following revenues and expenditures:

**Revenues**	**Expenditures**
Federal Income Tax	OASI benefits
State and City Income Taxes	SSDI benefits
Social Security Payroll Tax	SSI benefits
Medicare Payroll Tax	Medical Care Costs
Property Tax	Medicaid
	Medicare (parts A, B, and D)

6.1 Old-Age and Survivors Insurance benefits

Workers with 40 quarters of coverage and of age 62 are eligible to receive their retirement benefit. The benefit is calculated based on the Average Indexed Monthly Earnings (AIME) and the age at which benefits are first received. If an individual claims at his NRA (65 for those born prior to 1943, 66 for those between 1943 and 1957, and 67 thereafter), he receives his Primary Insurance Amount (PIA) as a monthly benefit. The PIA is a piece-wise linear function of the AIME. If a worker claims prior to his NRA, his benefit is lower than his PIA. If he retires after the NRA, his benefit is higher. While receiving benefits before reaching NRA, earnings are taxed above a certain earning disregard level. An individual is eligible to half of his spouse's PIA, properly adjusted for the claiming age, if that is higher than his/her own retirement benefit. A surviving spouse is eligible to the deceased spouses PIA. Since we assume prices are constant in our simulations, we do not adjust benefits for the Cost of Living Adjustment (COLA) which usually follows inflation. We however adjust the PIA bend points for increases in real wages.

6.2 Social Security Disability Insurance benefits

Workers with enough quarters of coverage and under the normal retirement age are eligible for their PIA (no reduction factor) if they are judged disabled (which we take as the predicted outcome of Social Security Disability Insurance (SSDI) receipt) and earnings are under a cap called the Substantial Gainful Activity (SGA) limit. This limit was $9720 in 2004. We ignore the 9 month trial period over a 5 year window in which the SGA is ignored.

6.3 Supplemental Security Income benefits

Self-reported receipt of Supplemental Security Income (SSI) in the HRS provides estimates of the proportion of people receiving SSI under what other estimates would suggest. To correct for this bias, we use a probit of receiving SSI as a function of self-reporting social security income, as well as demographic, health, and wealth. This probit is adjusted to target a 4% claiming rate.

The benefit amount is taken from the average monthly benefits found in the 2004 Social Security Annual Statistical Supplement. We assign monthly benefit of $450 for person aged 51 to 64, and $350 for persons aged 65 and older.

6.4 Medical costs estimation

In THEMIS, a cost module links a person's current state–demographics, economic status, current health, risk factors, and functional status to 4 types of individual medical spending. THEMIS models: total medical spending (medical spending from all payment sources), Medicare spending,[6] Medicaid spending (medical spending paid by Medicaid), and Out-of-Pocket (OOP) spending (medical spending by the respondent). These estimates are based on pooled weighted OLS regressions of each type of spending on risk factors, self-reported conditions, and functional status, with spending inflated to constant dollars using the medical component of the Consumer Price Index (CPI). We use the 2000-2010 MEPS for these regressions for persons not Medicare eligible, and the 2000-2010 MCBS for spending for those that are eligible for Medicare. Those eligible for Medicare include people eligible due to age (65+) or due to disability status. Comparisons of prevalences and question wording across these different sources are provided in Tables 1 and 2, respectively.

In the baseline scenario, this spending estimate can be interpreted as the resources consumed by the individual given the manner in which medicine is practiced in the US during the post-Part D era (2006-2010). Models are estimated for total, Medicaid, OOP spending, and for the Medicare spending. These estimates only use the MCBS dataset.

Since Medicare spending has numerous components (Parts A and B are considered here), models are needed to predict enrollment. In 2004, 98.4% of all Medicare enrollees, and 99%+ of aged enrollees, were in Medicare Part A, and thus we assume that all persons eligible for Medicare take Part A. We use the 2007-2010 MCBS to model take up of Medicare Part B for both new enrollees into Medicare, as well as current enrollees without Part B. Estimates are based on weighted probit regression on various risk factors, demographic, and economic conditions. The HRS starting population for THEMIS does not contain information on Medicare enrollment. Therefore another model of Part B enrollment for all persons eligible for Medicare is estimated via a probit, and used in the first year of simulation to assign initial Part B enrollment status. The MCBS data overrepresents the portion of eligible adults enrolled in Part B, having a 97% enrollment rate in 2004 instead of the

[6]We estimate annual medical spending paid by specific parts of Medicare (Parts A, B, and D) and sum to get the total Medicare expenditures.

93.5% rate given by Medicare Trustee's Report (30). In addition to this baseline enrollment probit, we apply an elasticity to premiums of -0.10, based on the literature and simulation calibration for actual uptake through 2009 (1; 4). The premiums are computed using average Part B costs from the previous time step and the means-testing thresholds established by the ACA.

Since both the MEPS and MCBS are known to under-predict medical spending (see, e.g., Selden and Sing, 2008, and references therein), we applied adjustment factors to the predicted three types of individual medical spending so that the predicted per-capita spending in THEMIS equals the corresponding spending in the National Health Expenditure Accounts (NHEA) for age group 55-64 in year 2004 and age 65+ in year 2010, respectively. Table W shows how these adjustment factors were determined by using the ratio of expenditures in the NHEA to expenditures predicted in THEMIS.

Since 2006, the MCBS has contained data on Medicare Part D. The data gives the capitated Part D payment and enrollment. When compared to the summary data presented in the CMS 2007 Trustee Report (31), the 2006 per capita cost is comparable between the MCBS and CMS. However, the enrollment is underestimated in the MCBS, 53% compared to 64.6% according to CMS.

A cross-sectional probit model is estimated using years 2007-2010 to link demographics, economic status, current health, and functional status to Part D enrollment. To account for both the initial underreporting of Part D enrollment in the MCBS, as well as the CMS prediction that Part D enrollment will rise to 75% by 2012, the constant in the probit model is increased by 0.22 in 2006, to 0.56 in 2012 and beyond.[7] The per capita Part D cost in the MCBS matches well with the cost reported from CMS. An OLS regression using demographics, current health, and functional status is estimated for Part D costs based on capitated payment amounts.

The Part D enrollment and cost models are implemented in the Medical Cost module. The Part D enrollment model is executed conditional on the person being eligible for Medicare, and the cost model is executed conditional on the enrollment model leading to a true result, after the Monte Carlo decision. Otherwise the person has zero Part D cost. The estimated Part D costs are added to Part A and B costs to obtain total Medicare cost, and any medical cost growth assumptions are then applied.

6.5 Taxes

We consider Federal, State and City taxes paid at the household level. We also calculate Social Security taxes and Medicare taxes. HRS respondents are linked to their spouse in the HRS simulation. We take program rules from the Organization for Economic Co-operation and Development (OECD) Taxing Wages Publication for 2004 (OECD). Households have basic and personal deductions based on marital status and age (>65). Couples are assumed to file jointly. Social Security benefits are partially taxed. The amount taxable increases with other income from 50% to 85%. Low income elderly have access to a special tax credit and the earned income tax credit is applied for individuals younger than age 65. We calculate state and city taxes for someone living in Detroit, Michigan. The OECD chose this location because it is generally representative of average state and city taxes paid in the US.

At the state level, there is a basic deduction for each member of the household ($3,100) and taxable income is taxed at a flat rate of 4%. At the city level, there is a small deduction of $750 per household member and the remainder is taxed at a rate of 2.55%. There is however a tax credit

[7] There is now enough data to estimate these models directly and this effort is underway

that decreases with income (20% on the first 100$ of taxes paid, 10% on the following 50$ and 5% on the remaining portion).

We calculate taxes paid by the employee for Social Security (Old-Age and Survivors Insurance (OASI) and SSDI) and Medicare (Medicaid and Medicare). It does not include the equivalent portion paid by the employer. OASI taxes of 6.2% are levied on earnings up to $97,500 (2004 cap) while the Medicare tax (1.45%) is applied to all earnings.

7 Scenarios and robustness

7.1 Obesity reduction scenario

In addition the to the status quo scenario, THEMIS can be used to estimate the effects of numerous possible policy changes. One such set of policy simulations involves changing the trends of risk factors for chronic conditions. This is implemented by altering the incoming cohorts. An useful example is an obesity reduction scenario which rolls back the prevalence of obesity among 50 year-olds to its 1978 level by 2030, where it remains until the end of the scenario, in 2050. This is accomplished by reversing the annual rates of change for Body Mass Index (BMI) category, hypertension, and diabetes shown in Table E. As seen in Table Y, this will change the prevalence of obesity among those aged 50+ in 2050. As compared with the status quo estimates (Table X), THEMIS predicts that by 2050 this will result in a change in the amount of Social Security benefits as well as changing combined Medicare and Medicaid expenditures.

8 Implementation

THEMIS is implemented in multiple parts. Estimation of the transition and cross sectional models is performed in Stata. The incoming cohort model is estimated in Stata using the CMP package (26). The simulation is implemented in C++ to increase speed.

To match the two year structure of the HRS data used to estimate the transition models, THEMIS proceeds in two year increments. The end of each two year step is designed to occur on July 1st to allow for easier matching to population forecasts from Social Security. A simulation of THEMIS proceeds by first loading a population representative of the age 51+ U.S. population in 2004, generated from HRS. In two year increments, THEMIS applies the transition models for mortality, health, working, wealth, earnings, and benefit claiming with Monte Carlo decisions to calculate the new states of the population. The population is also adjusted by immigration forecasts from the U.S. Census Department, stratified by race and age. If incoming cohorts are being used, the new 51/52 year olds are added to the population. The number of new 51/52 year-olds added is consistent with estimates from the Census, stratified by race. Once the new states have been determined and new 51/52 year-olds added, the cross sectional models for medical costs, and calculations for government expenditures and revenues are performed. Summary variables are then computed. Computation of medical costs includes the persons that died to account for end of life costs. Other computations, such as Social Security benefits and government tax revenues, are restricted to persons alive at the end of each two year interval. To eliminate uncertainty due to the Monte Carlo decision rules, the simulation is performed multiple times (typically 100), and the mean of each summary variable is calculated across repetitions.

THEMIS takes as inputs assumptions regarding growth in the National Wage Index, NRA, real medical cost growth, interest rates, COLA, the CPI, SGA, and deferred retirement credit.

The default assumptions are taken from the 2010 SSI Intermediate scenario, adjusted for no price increases after 2010. Therefore simulation results are in real 2009 dollars. Table Z shows the assumptions for each calendar year and Table AA shows assumptions for each birth year.

Different simulation scenarios are implemented by changing any of the following components: incoming cohort model, transition models, interventions that adjust the probabilities of specific transition, and changes to assumptions on future economic conditions.

8.1 Intervention module

The intervention module can adjust characteristics of individuals when they are first read into the simulation (initial interventions) or alter transitions within the simulation (transition interventions). At present, inital interventions can act on chronic diseases, ADL and IADL limitation status, program participation, and some demographic characteristics. Transition interventions can currently act on mortality, chronic diseases, and some program participation variables.

Transition interventions can take several forms. The most commonly used is an adjustment to a transition probability. One can also delay the assignment of a chronic condition or cure an existing chronic condition. Additional flexibility comes from selecting who is eligible for the intervention. Some examples might help to make the interventions concrete:

- Example 1: Delay the enrollment into OASI by two years. In this scenario claiming of Social Security benefits is transitioned as normal. However, if a person is predicted to claim their benefits, then that status is not immediately assigned, but is instead assigned two years later.

- Example 2: Cure hypertension for those with no other chronic diseases. In this scenario any individual with hypertension (including those who have had hypertension for many years) is cured (hypertension status is set to 0), as long as they do not have other chronic diseases. This example uses the individuals chronic disease status as the eligibility criteria for the intervention.

- Example 3: Reduce the incidence of hypertension for half of men aged 55 to 65 by 10% in the first year of the simulation, gradually increasing the reduction to 20% after 10 years. This example begins to show the flexibility in the intervention module. The eligibility criteria are more complex (half of men in a specific age range are eligible) and the intervention changes over time. Mathematically, the intervention works by acting on the incidence probability, ρ. In the first year of the simulation, the probability is replaced by $(1 - 0.5 * 0.1)\,\rho = 0.95\rho$. The binary outcome is then assigned based on this new probability. Thus, at the population level, there is a 5% reduction in incidence for men aged 55 to 65, as desired. After 10 years, the probability for this eligible population becomes $(1 - 0.5 * 0.2)\,\rho = 0.9\rho$.

More elaborate interventions can be programmed by the user.

9 Model development

This section gives some historical background about decisions and developments that led up to the current state of THEMIS.

9.1 Transition model

Section 4.1 describes the current THEMIS transition model with a focus on discrete absorbing outcomes. In developing this model, it was previously assumed that the time invariant part of the hazard was composed of the effect of observed characteristics x_i and permanent unobserved characteristics specific to outcome m, $\eta_{i,m}$. Consequently, the index was assumed to be of the form $z_{m,j_i} = x_i\beta_m + h_{i,j_i-1,-m}\gamma_m + \eta_{i,m}$ and the latent component of the hazard was modeled as

$$h^*_{i,j_i,m} = x_i\beta_m + h_{i,j_i-1,-m}\gamma_m + \eta_{i,m} + a_{m,j_i} + \varepsilon_{i,j_i,m}, \tag{6}$$

$$m = 1, \ldots, M_0,\ j_i = j_{i0}, \ldots, j_{i,T_i},\ i = 1, \ldots, N$$

This is the same as Equation (1), except that Equation (6) uses unobserved characteristics $\eta_{i,m}$ instead of the effects of observed initial conditions $h_{i,j_0,-m}\psi_m$. The unobserved effects $\eta_{i,m}$ are persistent over time and were allowed to be correlated across diseases $m = 1, \ldots, M$. We assumed that these effects had a normal distribution with covariance matrix $\mathbf{\Omega}_\eta$.

The parameters $\theta_1 = \left(\{\beta_m, \gamma_m, \varsigma_m\}_{m=1}^{M}, \text{vech}(\mathbf{\Omega}_\eta)\right)$ could be estimated by maximum simulated likelihood. The joint probability, conditional on the individual frailty is the product of normal univariate probabilities. Similar to the joint probability in Section 4.1, these sequences, conditional on unobserved heterogeneity, are also independent across diseases. The joint probability over all disease-specific sequences is simply the product of those probabilities.

For a given respondent with frailty η_i, the probability of the observed health history is (again, omitting the conditioning on covariates for simplicity)

$$l_i^{-0}(\theta; \eta_i, h_{i,j_{i0}}) = \left[\prod_{m=1}^{M-1}\prod_{j=j_{i1}}^{j_{T_i}} P_{ij,m}(\theta;\eta_i)^{(1-h_{ij-1,m})(1-h_{ij,M})}\right] \times \left[\prod_{j=j_{i1}}^{j_{T_i}} P_{ij,M}(\theta;\eta_i)\right]$$

To obtain the likelihood of the parameters given the observables, it is necessary to integrate out unobserved heterogeneity. The complication is that $h_{i,j_{i0},-m}$, the initial outcomes in each hazard, are not likely to be independent of the common unobserved heterogeneity term which needs to be integrated out. A solution is to model the conditional probability distribution $p(\eta_i \mid \mathbf{h}_{i,j_{i0}})$ (34). Implementing this solution amounts to including initial outcomes at baseline (age 50) for each hazard. This is equivalent to writing

$$\eta_i = \Gamma h_{i0} + \alpha_i$$
$$\alpha_i \sim \mathrm{N}(0, \Omega_\alpha)$$

Therefore, this allows for permanent differences in outcomes due to differences in baseline outcomes. The likelihood contribution for one respondent's sequence is therefore given by

$$l_i(\theta, \mathbf{h}_{i,j_{i0}}) = \int l_i(\theta; \alpha_i, \mathbf{h}_{i,j_{i0}})dF(\alpha_i) \tag{7}$$

This model was estimated using maximum simulated likelihood. The likelihood contribution Equation (7) was replaced with a simulated counterpart based on R draws from the distribution of α. The Broyden-Fletcher-Goldfarb-Shanno algorithm was then used to optimize over this simulated likelihood. Convergence of the joint estimator could not be obtained, so the distribution of α_i was assumed to be degenerate. This yielded the simpler estimation problem describe in Section 4.1, where each equation is estimated separately.

9.2 Quality-adjusted life years

9.2.1 Health-related quality-of-life

In general, HRQoL measures summarize population health by a single preference-based index measure. A HRQoL measure is a suitable measure of a QALY. There are several widely-used generic HRQoL indexes, each involving a standard descriptive system: a multidimensional measure of health states and a corresponding scoring system to translate the descriptive system into a single index (8). The scoring system is developed based on a community survey of preference valuation of health states in the descriptive system, using utility valuation methods like time trade-offs or a standard gamble.

9.2.2 HRQoL in the MEPS

Because the health states measures in the HRS and THEMIS do not match the health states defined in any of the currently available HRQoL indexes, we used the MEPS to create a crosswalk file for HRQoL index calculation. The MEPS collects information on health care cost and utilization, demographics, functional status, and medical conditions. Since the year 2000, it initiated a self-administered questionnaire for two sets of instruments: 12-Item Short Form Health Survey (SF-12) and EQ-5D.

Seven of the twelve SF-12 questions can be used to generate another HRQoL index: Short-Form 6D (SF-6D). However, the scoring system for SF-6D was derived from a UK sample (3), and a significant proportion of the MEPS sample do not give valid answers for at least one of the seven questions. Therefore, we calculate the EQ-5D index score as the HRQoL measure for THEMIS.

The EQ-5D instrument includes 5 questions about the extent of problems in mobility, self-care, daily activities, pain, and anxiety/depression. The scoring system for EQ-5D was first developed by Dolan (7) using a UK sample. Later, a scoring system based on a US sample was generated (29). In the MEPS 2001, there are 8,301 respondents aged 51 and over. Of those respondents, 7,439 gave valid answers for all of the five EQ-5D questions. We calculate EQ-5D scores for these respondents using the scoring algorithm based on a US sample (29). The distribution of EQ-5D index scores among these respondents is shown in Figure B

9.2.3 MEPS-HRS crosswalk development

The functional status measure in THEMIS is based on the HRS. It is a categorical variable including the following mutually exclusive categories: healthy, any IADL limitations (no ADL limitations), 1-2 ADL limitations, and 3 or more ADL limitations. Unfortunately the measures of IADL and ADL limitations in the MEPS are quite different. The HRS asks questions like "Do you have any difficulty in . . .", while the MEPS asks questions like "Does . . .help or supervision in . . .." As Table O shows, the prevalence of IADL limitations is relatively similar between the two surveys, while the prevalence of ADL limitations is much higher in the HRS, relative to the MEPS. This is reasonable since not all who have ADL limitations receive help or supervision.

In order to compute EQ-5D index scores using functional status in THEMIS, we need a set of functional status measures that is comparable across the MEPS and the HRS (the host dataset for THEMIS). We explore several options for deriving such a measure. Ultimately, we construct two measures. One measure indicates physical function limitations while the other measure indicates IADL limitations.

In the MEPS, physical function limitation indicates that at least one of the following is true: 1) receiving help or supervision with bathing, dressing or walking around the house; 2) being limited in

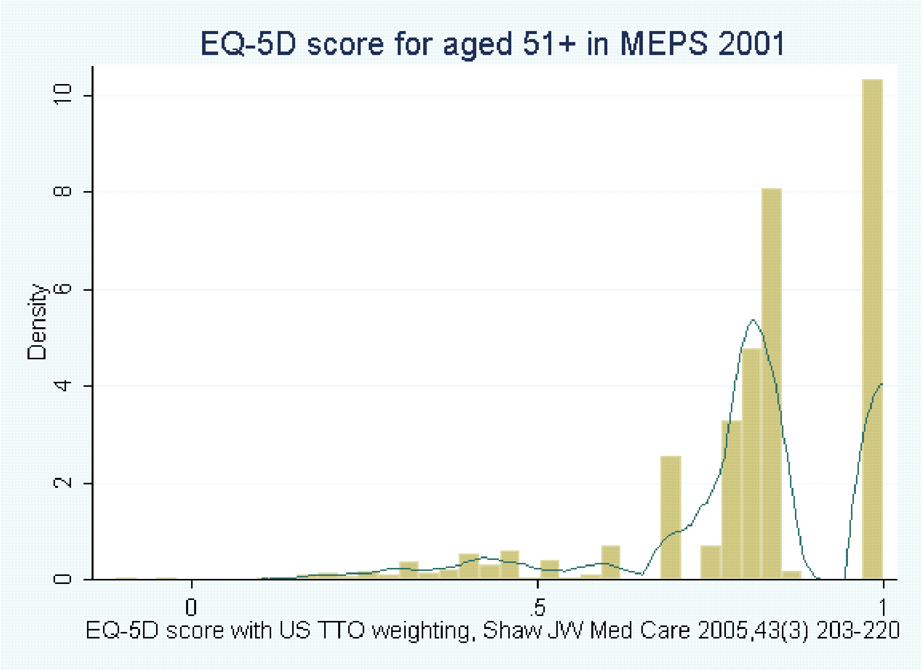

Figure B: Distribution of EQ-5D index scores for ages 51+ in the 2001 MEPS

work/housework; 3) having difficulty walking, climbing stairs, grasping objects, reaching overhead, lifting, bending or stooping, or standing for long periods of time; or 4) having difficulty in hearing or vision. In the HRS, physical function limitation indicates that at least one of the following is true: 1) having any difficulty in bathing/dressing/eating/walking across the room/getting out of bed; 2) limited in work/housework; or 3) limited in any other activities.

In the MEPS, IADL limitation indicates receiving help or supervision using the telephone, paying bills, taking medications, preparing light meals, doing laundry, or going shopping. In the HRS, IADL limitation indicates having difficulty in any IADL such as using the phone, managing money, or taking medications.

The prevalence of our two constructed measures among those aged 51 and older in the MEPS (2001) and the HRS (1998) is shown in Table P. The prevalences are quite similar across the two surveys.

Using the MEPS 2001 data, we next use OLS regression to model the derived EQ-5D score as a function of six chronic conditions – which are available in both the HRS and the MEPS, our two constructed measures of functional status, and an interaction term of the two measures of functional status. Three different models are considered. Estimation results are presented in Models I-III in Table Q. We also show the estimation results of using only IADL/ADL limitation as covariates, and using only the six chronic conditions as covariates, as Models IV and V in Table Q. Model II was used as the crosswalk described in Section 4.2 to calculate EQ-5D score for non-nursing home residents aged 51 and over in the HRS data for 1998.

9.3 Drug expenditures

9.3.1 Drug expenditures - MEPS

Agency for Healthcare Research and Quality produces a file of consolidated annual expenditures for each MEPS respondent in each calendar year. The total drug expenditure variable sums all

amounts paid OOP and by third party payers for each prescription purchased in that year. For comparison across years, we convert all amounts to 2016 dollars using the Medical CPI.

9.3.2 Drug expenditures - MCBS

The MCBS produces a Prescribed Medicine Events file at the individual-event level, with cost and utilization of prescribed medicines for the MCBS community population. Collapsing this file to the individual provides an estimate of prescription drug cost for each person. For comparison across years, we convert all amounts to 2016 dollars using the Medical CPI.

There are two caveats to working with these data. The first caveat regards how to handle the ”ghost” respondents. ”Ghosts” are individuals who enroll in Medicare, but were not asked cost and use questions in the year of their enrollment. For some outcomes, such as medical expenditures, the MCBS makes an effort to impute. For others, such as drug utilization and expenditures, the MCBS does not. We imputed annual drug expenditures for the ”ghosts”, but for certain age ranges the drug expenditures were not reasonable. This had the biggest effect on the 65/66 year olds, for two reasons. The first is that the 65/66 year olds are more likely to be ”ghosts”, as 65 is the typical age of enrollment for Medicare. The second is that the 65/66 year olds used for imputation (i.e., the non-”ghosts”) are different. To be fully present in the MCBS at age 65 would require enrolling in Medicare before age 65, which happen through a different channel, such as qualifying for Medicare due to receiving disability benefits from the federal government.

The second caveat relates to the filling in zeroes for individuals with no utilization data, but who were enrolled. We assumed that individuals who were not ”ghosts” and who did not appear on the Prescribed Medicine Events file had zero prescription expenditures.

9.3.3 Drug expenditures - estimation

Due to the complexities of the age 65/66 population in the MCBS, we chose to estimate the drug expenditure models using the MEPS for individuals aged 51 to 66 and the MCBS for individuals aged 67 and older. Individuals under age 65 receiving Medicare due to disability are estimated separately. Since there are a number of individuals with zero expenditures, we estimate the models in two stages. The first stage is a probit predicting any drug expenditures and the second is an OLS model predicting the amount, conditional on any. Coefficient estimates and marginal effects are shown in the accompanying Excel workbook.

10 Validation

We perform three validation exercises:

1. Cross-validation
2. External validation
3. External corroboration

Cross-validation is a test of the simulation’s internal validity that compares simulated outcomes to actual outcomes, external validation compares model forecasts with actual outcomes from other data sources, and external corroboration compares model forecasts to others’ forecasts.

10.1 Cross-validation

The cross-validation exercise randomly samples half of the HRS respondent identifiers for use in estimating the transition models. The respondents not used for estimation, but who were present in the HRS sample in 1998, are then simulated from 1998 through 2012. Demographic, health, and economic outcomes are compared between the simulated (THEMIS) and actual (HRS) populations. These results are presented in Table I - Table N for 2000, 2006, and 2012, with a statistical test of the difference between the average values in the two populations.

Worth noting is how the composition of the population changes in this exercise. In 1998, the sample represents those aged 51 and older. Since we follow a fixed cohort, the age of the population will increase to 65 and older in 2012. This has consequences for some measures in later years where the eligible population shrinks.

On the whole, the cross-validation exercise is reassuring. Comparing simulated outcomes to actual outcomes using a set of transition models estimated on a separate population reveals that the majority of outcomes of interest are not statistically different. In cases where they are, the practical difference is potentially low.

10.2 External validation

The external validation exercise compares THEMIS full population simulations beginning in 2004 to external sources. Here we focus on per capita benefits received from the OASI, SSDI, and SSI, followed by Medicare and Medicaid.

10.2.1 Benefits from Social Security Administration

Conditional on a simulant receiving benefits, THEMIS algorithmically assigns benefits for OASI, SSDI, SSI. Here we compare simulation results to SSA figures.

For the OASI benefits, we compare to the SSA December 2012 Monthly Statistical Snapshot. Table B of that document indicates that the average OASI monthly benefit was $1194. THEMIS forecasts $1182 for the average beneficiary for 2012.

For the SSI, we compare to Table C of the December 2012 Monthly Statistical Snapshot, focusing on the population aged 65 and older, as that is the only category that is directly comparable. The SSA reports that the average monthly benefit for December of 2012 was $417. THEMIS assigns$415 to those receiving SSI.

The SSA does not report SSDI figures that are directly comparable to THEMIS forecasts. However, the SSA reports average SSDI benefits by age, as well as the number of individuals receiving benefits at each age. This allows us to construct the average benefit for workers 51 and older. Based on this calculation, the average disabled worker aged 51+ received a benefit of $1212 in December of 2012. Spouses of disabled workers can also receive a benefit (SSA reports a benefit of $304 for spouses of disabled workers for all ages). The 2012 THEMIS forecast for the average SSDI beneficiary, which includes both workers and their spouses, is $1102.

10.2.2 Benefits from Medicare and Medicaid

For medical spending, we compare THEMIS forecasts in 2010 to the NHEA measures from 2010, the most recent year for which these data are available. NHEA reports total amounts by age range, which we then convert to per capita measures using the 2010 Census. We focus on the 65-84 and 85 plus age groups, as they are directly comparable to THEMIS forecasts. We also aggregate

the two groups to produce a 65 plus average. THEMIS is closest to the NHEA for the Medicare and total medical spending measures. THEMIS estimates are less accurate for Medicaid spending. These adjustment factors are then used in the simulation results to more closely match the NHEA numbers.

10.3 External corroboration

Finally, we compare THEMIS population forecasts to Census forecasts of the U.S. population. Here, we focus on the full HRS population (aged 51 and older) and those aged 65 and older. For this exercise, we begin the simulation in 2010 and simulate the full population through 2050. Population projections are compared to the 2012 Census projections for years 2012 through 2050. THEMIS population forecasts are always within two percent of Census forecasts.

11 Baseline forecasts

In this section we present baseline forecasts of THEMIS. The figures show data from the HRS for the 55+ population from 1998 through 2012 and forecasts from THEMIS for the 55+ population beginning in 2010.

11.1 Disease prevalence

Figure C depicts the six chronic conditions we project for men. And Figure D depicts the historic and forecasted values for women.

Figure E: shows historic and forecasted levels for any ADL limitations, three or more ADL limitations, any IADL limitations, and two or more IADL limitations for men 55 and older. Figure F shows historic and forecasted levels for any ADL limitations, three or more ADL limitations, any IADL limitations, and two or more IADL limitations for women 55 and older.

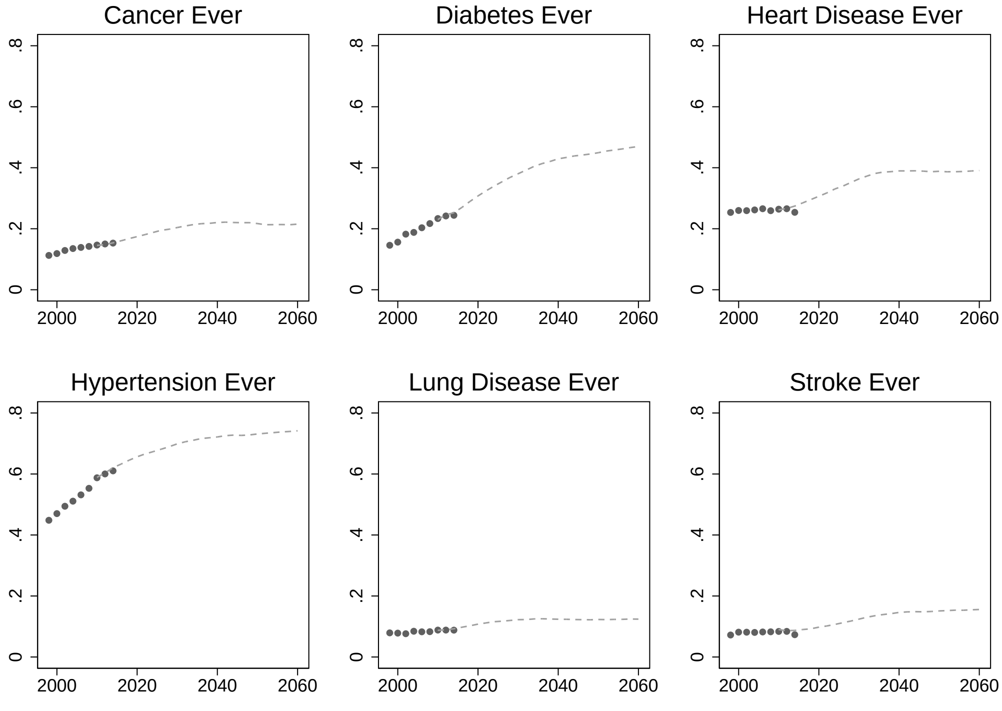

Figure C: Historic and forecasted chronic disease prevalence for men 55+

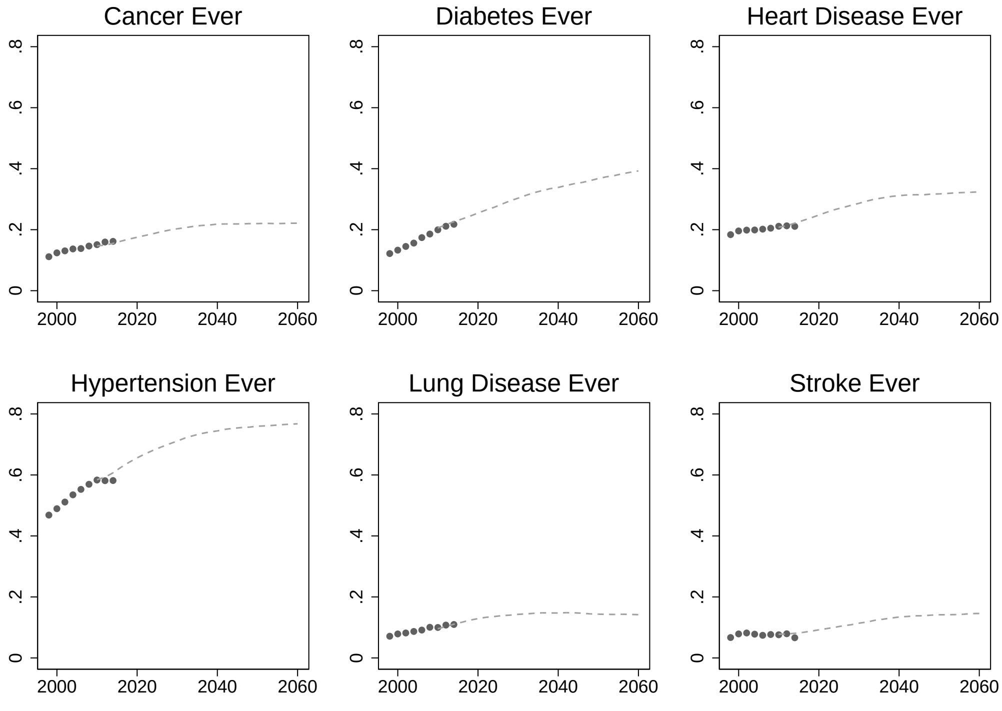

Figure D: Historic and forecasted chronic disease prevalence for women 55+

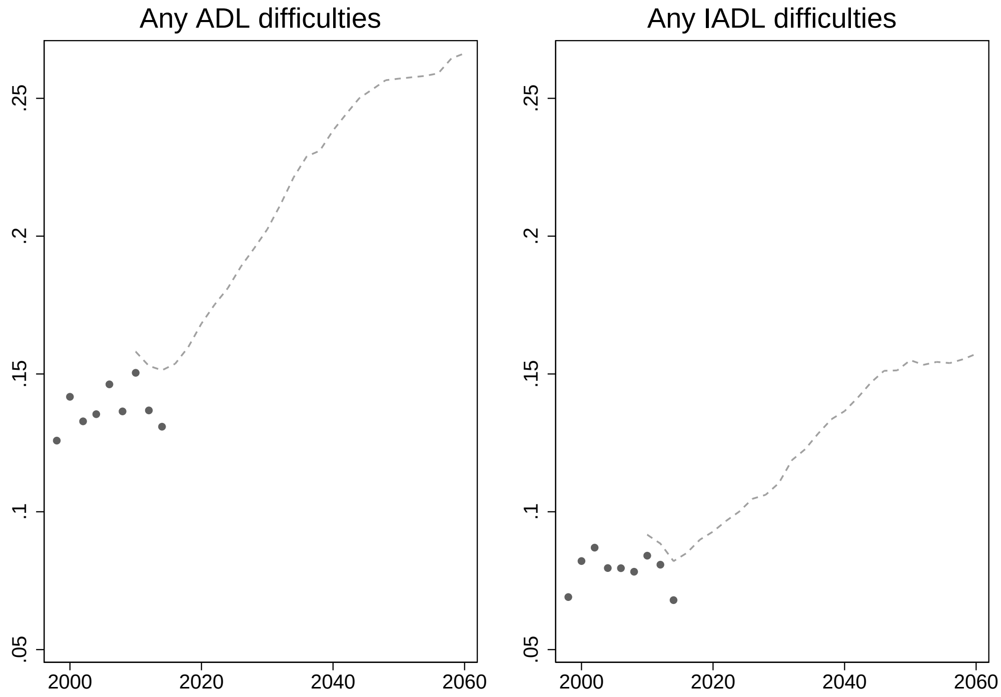

Figure E: Historic and forecasted ADL and IADL prevalence for men 55+

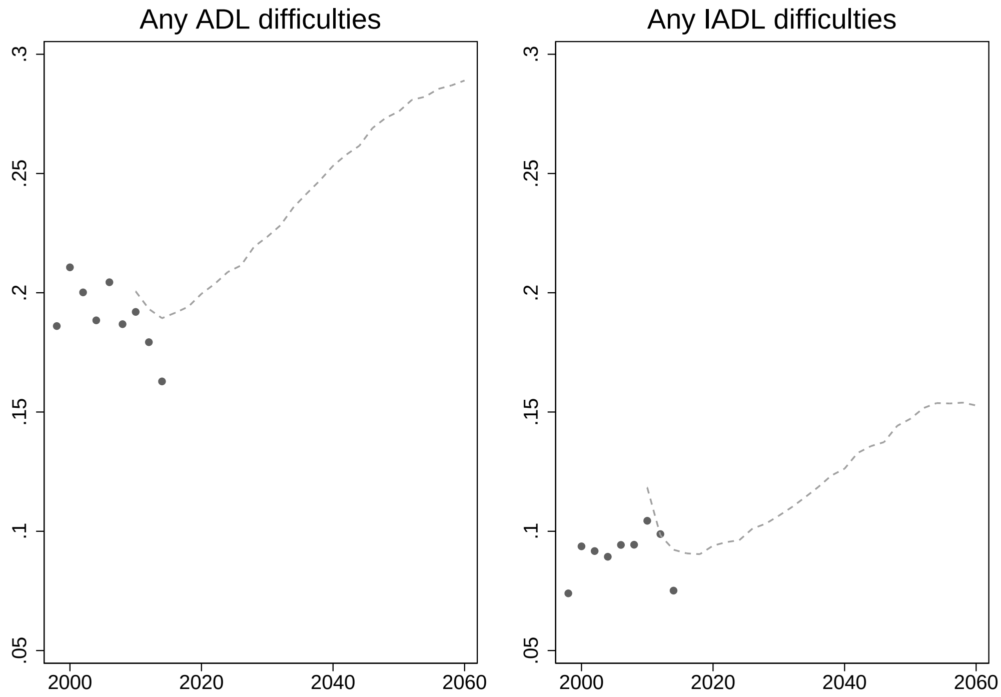

Figure F: Historic and forecasted ADL and IADL prevalence for women 55+

12 Tables

					Prevalence %			
Source (years, ages)	Cancer	Heart Diseases	Stroke	Diabetes	Hypertension	Lung Disease	Overweight	Obese
HRS (1991-2008, 55-64)	8%	14%	4%	15%	44%	7%	39%	33%
NHIS (1997-2010, 55-64)	8%	17%	4%	13%	43%	8%	37%	33%
MEPS (2000-2010, 55-64)	7%	17%	4%	15%	47%	7%	38%	32%
HRS (1991-2008, 65+)	18%	30%	11%	20%	59%	11%	38%	23%
NHIS (1997-2010, 65+)	16%	31%	9%	16%	56%	10%	36%	24%
MCBS (2000-2010, 65+)	18%	41%	11%	23%	65%	16%	38%	23%
MEPS (2000-2010, 65+)	12%	33%	11%	19%	64%	10%	38%	25%

Table A: Health condition prevalences in survey data

	Survey			
Disease	HRS	NHIS	MEPS	MCBS
Cancer	Has a doctor ever told you that you have cancer or a malignant tumor, excluding minor skin cancers?	Have you ever been told be a doctor or other health professional that you had cancer or a malignanacy of any kind? (WHEN RECODED, SKIN CANCERS WERE EXCLUDED)	List all the conditions that have bothered (the person) from (START time) to (END time) CCS codes for the conditions list are 11-21, 24-45	Has a doctor ever told you that you had any (other) kind of cancer malignancy, or tumor other than skin cancer?
Heart Diseases	Has a doctor ever told you that you had a heart attack, coronary heart disease, angina, congestive heart failure, or other heart problems?	Four separate questions were asked about whether ever told by a docotor or oither health professional that had: CHD, Angina, MI, other heart problems.	Have you ever been told by a doctor or health professional that you have CHD; Angina; MI; other heart problems	Six separate questions were asked about whether ever told by a doctor that had: Angina or MI; CHD; other heart problems (included four questions)
Stroke	Has a doctor ever told you that you had a stroke?	Have you EVER been told by a doctor or other health professional that you had a stroke?	If Female, add: [Other than during pregnancy,] Have you ever been told by a doctor or health professional that you have a stroke or TIA (transient ischemic attack)	[Since (PREV< SUPP. RD. INT. DATE),] has a doctor (ever) told (you/SP) that (you/he/she) had a stroke, a brain hemorrhage, or a cerebrovascular accident?
Diabetes	Has a doctor ever told you that you have diabetes or high blood sugar?	If Female, add: [Other than during pregnancy,] Have you ever been told by a doctor or health professional that you have diabetes or sugar diabetes?	If Female, add: [Other than during pregnancy,] Have you hever been told by a doctor or health professional that you have diabetes or sugar diabetes?	Has a doctor (ever) told (you/SP) that (you/he/she) had diabetes, high blood sugar, or sugar in (your/his/her) urine? [DO NOT INCLUDE BOERDERLINE PREGNANCY, OR PREDIABETEIC DIABETES.]
Hypertension	Has a doctor ver told you that you have high blood pressure or hypertension?	Have you EVER been told by a doctor or other health professional that you had Hypertension, also called high blood pressure?	Have you EVER been told by a doctor or other health professional that you had Hypertension, also called high blood pressure?	Has a doctor (ever) told (you/SP) that (you/he/she) (still) (had) (have/has) hypertension, sometimes called high blood pressure?
Lung Disease	Has a doctor ever told you that you have chronic lung disease such a schronic bronchitis or emphysema? [IWER: DO NOT INCLUDE ASTHMA]	Question 1: During the PAST 12 MONTHS, have you ever been told by a doctor or other health professional that you had chronic bronchitis? Question 2: Have you EVER been told by a docotor or other health professional that you had emphysema?	List all the conditions that have bothered (the person) from (START time) to (END time) CCS codes for the conditions list are 127, 129-312	Has a doctor (ever) told (you/SP) that (you/he/she) had emphysema, asthma, or COPD? [COPD=CHRONIC OBSTRUCTIVE PULMONARY DISEASE.]
Overweight	Self-reported body weight and height			
Obese				

Table B: Survey questions used to determine health conditions

Conditions	Data source	Projection method	Other sources
Diabetes<br>Heart disease<br>Hypertension	National Health Interview Survey 1997-2006	Use synthetic cohort approach to estimate age-specific incidence rate for each condition	There are other forecasts (14; 20) for the trends of diabetes in the U.S population; we compare their forecasts to ours and they are reasonably close
Overweight and obese	Prevalence of overweight and obese for aged 46-56 from year 2001 to 2030, generated by Ruhm upon request	Assume annual rate of change during year 2031-2050 linearly decreases from the 2030 rate to zero in 2050	Ruhm (27)
Ever-smoked and smoking now	Forecast of prevalence of ever-smoked and smoking now for aged 45-54 from year 2005 to 2025, by Levy (17)	For ever-smoked, assume that the prevalence at ages 45-54 in year 2035 (2045) is the same as prevalence at ages 35-44 (25-34) in year 2025. Assume that the annual change in prevalence at ages 45-54 in year 2046-2050 the same as average in 2040-2045. For smoking-now, after year 2025, use the moving average of the past five years	
Any DB from current job		Assume annual relative declining rate for DB entitlement decrease by 2% a year	Historical trends of DB participation rates among all persons by different birth cohorts and by age, by Poterba et al. (23)
Any DC from current job		Assume annual relative increasing rate for DC entitlement increase by 2% a year until 2026 then stays the same after 2026	Forecast of DC participation rates among all persons by different birth cohorts and by age, by Poterba et al. (24)
Population size 50-52<br>Male<br>Hispanic<br>Non-Hispanic black	Census Bureau 2000-2010 Intercensal Population Estimates, 2012 National Population Estimates, and 2012 National Population Projections	Projected 2060 - 2080 using linear trend based on 2040-2060	

Table C: Data sources and methods for projecting future cohort trends

Ratio of future prevalence to 2004 prevalence for 51/52 year olds

	Binary outcomes			Ordered outcomes (highest category)		Censored discrete outcomes	
Year	Hypertension	Heart Disease	Diabetes	BMI Status (BMI≥40)	Smoking Status (smoking now)	Any DB Plan	Any DC Plan
2010	1.00	1.00	1.00	1.00	1.00	1.00	1.00
2020	1.06	0.95	1.11	1.40	0.82	0.82	1.26
2030	1.08	0.93	1.14	1.81	0.66	0.67	1.41
2040	1.10	0.91	1.17	2.18	0.53	0.55	1.41
2050	1.12	0.90	1.20	2.30	0.41	0.45	1.41

Table D: Projected baseline trends for future cohorts

	Prevalence		
Condition	1978	2004	Annual rate of change to get 1978 prevalence by 2030
$30 \leq$ BMI < 35 (kg/m^2)	0.112	0.230	-0.027
$35 \leq$ BMI < 40 (kg/m^2)	0.028	0.060	-0.029
BMI >= 40 (kg/m^2)	0.014	0.040	-0.040
Hypertension	0.326	0.338	-0.001
Diabetes	0.047	0.103	-0.030
Currently smoking	0.398	0.281	0.013

Table E: Prevalence of obesity, hypertension, diabetes and current smokers among ages 46-56 in 1978 and 2004. Prevalence in 1978 is based on NHANES II 1976-1980; Prevalence in 2004 is based on NHANES 2003-2004. BMI is calculated using self-reported weight and height.

			Type	At risk	Mean/fraction
Disease	heart disease		biennial incidence	undiagnosed	0.03
	hypertension		biennial incidence	undiagnosed	0.04
	stroke		biennial incidence	undiagnosed	0.01
	lung disease		biennial incidence	undiagnosed	0.01
	cancer		biennial incidence	undiagnosed	0.02
	diabetes		biennial incidence	undiagnosed	0.02
Risk Factors	Smoking Status	never smoked	ordered	all	0.41
		ex smoker	ordered	all	0.43
		current smoker	ordered	all	0.16
	Log BMI		continuous	all	3.32
LFP & Benefits	working		prevalence	age < 80	0.49
	DB pension receipt		biennial incidence	eligible & not receiving	0.02
	SS benefit receipt		biennial incidence	eligible & not receiving	0.11
	DI benefit receipt		prevalence	eligible & age < 65	0.05
	Any health insurance		prevalence	age < 65	0.88
	SSI receipt		prevalence	all	0.03
	Nursing Home residency		prevalence	all	0.01
	Death		biennial incidence	all	0.04
Financial Resources ($USD 2004)	financial wealth		median	all non-zero wealth	187,667.72
	earnings		median	all working	18,599.70
	wealth positive		prevalence	all	0.97

Table F: Outcomes in the transition model. Estimation sample is HRS 1991-2008 waves.

Value at time $T-1$	Outcome at time T																			
	Heart disease	hypertension	stroke	Lung disease	diabetes	cancer	disability	mortality	Smoking status	BMI	Any HI	DI Claim	SS Claim	DB Claim	SSI Claim	Nursing Home	Work	Earnings	Nonzero Wealth	Wealth
Heart disease			✓				✓	✓	✓	✓	✓	✓	✓	✓	✓	✓	✓	✓	✓	✓
Blood pressure	✓		✓				✓	✓	✓	✓	✓	✓	✓	✓	✓	✓	✓	✓	✓	✓
Stroke							✓	✓	✓	✓	✓	✓	✓	✓	✓	✓	✓	✓	✓	✓
Lung disease							✓	✓	✓	✓	✓	✓	✓	✓	✓	✓	✓	✓	✓	✓
Diabetes	✓	✓	✓				✓	✓	✓	✓	✓	✓	✓	✓	✓	✓	✓	✓	✓	✓
Cancer			✓				✓	✓	✓	✓	✓	✓	✓	✓	✓	✓	✓	✓	✓	✓
Disability							✓	✓	✓	✓	✓	✓	✓	✓	✓	✓	✓	✓	✓	✓
Claimed DI											✓	✓	✓	✓	✓		✓	✓	✓	✓
Claimed SS											✓			✓	✓		✓	✓	✓	✓
Claimed DB													✓		✓		✓	✓	✓	✓
Claimed SSI															✓					
Work											✓	✓	✓		✓		✓	✓	✓	✓
Earnings											✓	✓	✓	✓	✓		✓	✓	✓	✓
Nonzero wealth											✓	✓	✓	✓	✓	✓	✓	✓	✓	✓
Wealth											✓	✓	✓	✓	✓	✓	✓	✓	✓	✓
Nursing home stay															✓	✓			✓	✓

Table G: Restrictions on transition model. ✓indicates that an outcome at time $T-1$ is allowed in the transition model for an outcome at time T.

	Unweighted Statistics			
Control variable	Mean	Standard deviation	Minimum	Maximum
Non-Hispanic black	0.141	0.348	0	1
Hispanic	0.0818	0.274	0	1
Less than high school	0.235	0.424	0	1
Some college and above	0.412	0.492	0	1
Male	0.416	0.493	0	1
Ever smoked	0.582	0.493	0	1
Fitted values	1839	1090	0	4273
frq				
Init.of Any DB from current job	0.277	0.448	0	1
fnra3	0.0427	0.202	0	1
fnra4	0.0309	0.173	0	1
fnra5	0.0612	0.240	0	1
Any DC from current job	0.124	0.329	0	1
(IHT of DC wlth in 1000s)/100 if any DC zero otherwise	0.00449	0.0133	0	0.0801

Table H: Desciptive statistics for exogeneous control variables in 2004 HRS ages 51+ sample used as simulation stock population

Outcome	2000 THEMIS mean	2000 HRS mean	2000 p	2006 THEMIS mean	2006 HRS mean	2006 p	2012 THEMIS mean	2012 HRS mean	2012 p
Died	0.100	0.048	0.000	0.062	0.078	0.000	0.080	0.086	0.155
Lives in nursing home	0.069	0.015	0.000	0.026	0.029	0.175	0.040	0.038	0.533

Table I: Crossvalidation of 1998 cohort: Simulated vs reported mortality and nursing home outcomes in 2000, 2006, and 2012

Outcome	2000 THEMIS mean	2000 HRS mean	2000 p	2006 THEMIS mean	2006 HRS mean	2006 p	2012 THEMIS mean	2012 HRS mean	2012 p
Age on July 1st	66.884	66.615	0.042	71.320	70.628	0.000	75.512	74.799	0.000
Black	0.086	0.092	0.121	0.083	0.086	0.407	0.080	0.086	0.242
Hispanic	0.060	0.055	0.070	0.061	0.056	0.106	0.063	0.059	0.376
Male	0.429	0.438	0.153	0.420	0.434	0.056	0.411	0.424	0.116

Table J: Crossvalidation of 1998 cohort: Simulated vs reported demographic outcomes in 2000, 2006, and 2012

Outcome	2000 THEMIS mean	2000 HRS mean	2000 p	2006 THEMIS mean	2006 HRS mean	2006 p	2012 THEMIS mean	2012 HRS mean	2012 p
Any ADLs	0.151	0.154	0.483	0.182	0.177	0.379	0.217	0.188	0.000
Any IADLs	0.074	0.068	0.070	0.091	0.089	0.726	0.109	0.112	0.647
Cancer	0.119	0.121	0.563	0.169	0.168	0.812	0.213	0.223	0.170
Diabetes	0.150	0.145	0.323	0.209	0.202	0.266	0.261	0.252	0.218
Heart Disease	0.207	0.210	0.493	0.277	0.271	0.395	0.347	0.336	0.191
Hypertension	0.477	0.462	0.022	0.600	0.591	0.241	0.693	0.679	0.089
Lung Disease	0.078	0.073	0.193	0.109	0.093	0.000	0.137	0.114	0.000
Stroke	0.065	0.069	0.275	0.095	0.098	0.521	0.126	0.119	0.216

Table K: Crossvalidation of 1998 cohort: Simulated vs reported binary health outcomes in 2000, 2006, and 2012

	2000			2006			2012		
	THEMIS	HRS		THEMIS	HRS		THEMIS	HRS	
Outcome	mean	mean	p	mean	mean	p	mean	mean	p
BMI	27.189	27.199	0.892	27.466	27.727	0.002	27.296	27.702	0.000
Current smoker	0.147	0.148	0.825	0.121	0.114	0.137	0.106	0.088	0.000
Ever smoked	0.582	0.592	0.134	0.572	0.582	0.149	0.555	0.566	0.199

Table L: Crossvalidation of 1998 cohort: Simulated vs reported risk factor outcomes in 2000, 2006, and 2012

	2000			2006			2012		
	THEMIS	HRS		THEMIS	HRS		THEMIS	HRS	
Outcome	mean	mean	p	mean	mean	p	mean	mean	p
Claiming DB pension	0.005	0.015	0.000	0.022	0.015	0.000	0.040	0.027	0.000
Claiming SSDI	0.064	0.041	0.000	0.066	0.085	0.019	0.037	0.129	0.000
Claiming OASI	0.809	0.794	0.100	0.748	0.767	0.064	0.899	0.865	0.004
Claiming SSI	0.029	0.045	0.000	0.017	0.037	0.000	0.012	0.030	0.000
Working for pay	0.446	0.443	0.691	0.346	0.355	0.244	0.245	0.242	0.751

Table M: Crossvalidation of 1998 cohort: Simulated vs reported binary economic outcomes in 2000, 2006, and 2012

	2000			2006			2012		
	THEMIS	HRS		THEMIS	HRS		THEMIS	HRS	
Outcome	mean	mean	p	mean	mean	p	mean	mean	p
Household wealth (thou.)	465	341.49	0	542.82	408.63	0	468.25	345.98	0
Capital income (thou.)	18.259	15.855	.01063	17.18	12.122	0	12.917	9.4581	.00082
Earnings (thou.)	14.808	16.804	0	10.856	12.646	.00013	7.2401	6.7241	.23917

Table N: Crossvalidation of 1998 cohort: Simulated vs reported continuous economic outcomes in 2000, 2006, and 2012

		MEPS 2001 (ages 51+) ADL limitation		
		No	Yes	All
IADL limitation	No	91.5	0.4	92.0
	Yes	4.4	3.7	8.0
	All	95.9	4.1	100.0

		HRS 1998 (ages 51+) ADL limitation		
		No	Yes	All
IADL limitation	No	82.0	10.5	92.5
	Yes	3.1	4.5	7.5
	All	85.1	14.9	100.0

Table O: Prevalence of IADL and ADL limitations among ages 51+ in the MEPS 2001 and the HRS 1998. The IADL limitations in MEPS are defined as receiving help or supervision using the telephone, paying bills, taking medications, preparing light meals, doing laundry, or going shopping; the ADL limitations in the HRS are defined as receiving help or supervision with personal care such as bathing, dressing, or getting around the house. The IADL limitations in the HRS are defined as having any difficulty in at least one of the following activities: using the phone, taking medications, and managing money. The ADL limitations in the HRS are defined as having any difficulty in at least one of the following activities: bathing, dressing, eating, walking across the room, and getting out of bed.

		MEPS 2001 (ages 51+) Physical function limitation		
		No	Yes	All
IADL limitation	No	61.6	30.4	92.0
	Yes	0.3	7.8	8.0
	All	61.9	38.2	100.0

		HRS 1998 (ages 51+) Physical function limitation		
		No	Yes	All
IADL limitation	No	60.0	32.5	92.5
	Yes	1.0	6.5	7.5
	All	61.0	39.0	100.0

Table P: Prevalence of IADL limitations and physical function limitations among ages 51+ in the MEPS 2001 and the HRS 1998. The definition of IADL limitation is the same as in Table O Physical function limitation in the MEPS indicates that at least one of the following is true: 1) receiving help or supervision with bathing, dressing or walking around the house; 2) being limited in work/ housework; 3) having difficulty walking, climbing stairs, grasping objects, reaching overhead, lifting, bending or stooping, or standing for long periods of time; or 4) having difficulty in hearing or vision. Physical function limitation in the HRS indicates at least one of the following is true: 1) having any difficulty in bathing/dressing/eating/walking across the room/getting out of bed; 2) limited in work/ housework; or 3) limited in any other activities.

	Model I	Model II	Model III	Model IV	Model V
Constant	0.877***	0.898***	0.874***	0.839***	0.869***
	(0.002)	(0.003)	(0.005)	(0.002)	(0.003)
Physical function limitation	-0.115***	-0.098***	-0.094***		
	(0.004)	(0.005)	(0.004)		
IADL limitation	-0.041	-0.019	-0.008		
	(0.037)	(0.042)	(0.036)		
IADL limitation * Physical function limitation	-0.150***	-0.156***	-0.162***		
	(0.037)	(0.044)	(0.037)		
IADL limitation, no ADL limitation				-0.182***	
				(0.009)	
Any ADL limitation				-0.344***	
				(0.010)	
Ever diagnosed with cancer		-0.011	-0.015**		-0.030***
		(0.009)	(0.007)		(0.010)
Ever diagnosed with diabetes		-0.034***	-0.032***		-0.054***
		(0.007)	(0.005)		(0.007)
Ever diagnosed with high blood pressure		-0.030***	-0.028***		-0.043***
		(0.004)	(0.004)		(0.005)
Ever diagnosed with heart disease		-0.024***	-0.029***		-0.055***
		(0.006)	(0.005)		(0.006)
Ever diagnosed with lung disease		-0.036***	-0.032***		-0.055***
		(0.009)	(0.007)		(0.010)
Ever diagnosed with stroke		-0.045***	-0.046***		-0.115***
		(0.012)	(0.008)		(0.013)
Age 65-74			0.010**		
			(0.004)		
Age 75 and over			0.015***		
			(0.005)		
Male			0.028***		
			(0.004)		
Non-Hispanic black			0.008		
			(0.007)		
Hispanic			-0.001		
			(0.007)		
Less than HS			-0.022***		
			(0.005)		
Some college			0.016***		
			(0.005)		
College grad			0.037***		
			(0.005)		
Census region: Northeast			0.003		
			(0.005)		
Census region: Midwest			0.004		
			(0.005)		
Census region: West			-0.012**		
			(0.005)		
Marital status:widowed			0.003		
			(0.005)		
Marital status: single			-0.013***		
			(0.005)		
N	7,358	7,317	7,317	7,361	7,322
Adjusted R^2	.24	.27	.29	.18	.11

* $p < 0.10$, ** $p < 0.05$, *** $p < 0.01$

Table Q: OLS regressions of EQ-5D utility index among ages 51+ in MEPS 2001. p-values in parentheses. Data source: MEPS 2001 (ages 51+). EQ-5D scoring algorithm is based on Shaw et al. (29).

Ever diagnosed with cancer	-0.020*
	(0.001)
Ever diagnosed with diabetes	-0.042*
	(0.001)
Ever diagnosed with heart disease	-0.044*
	(0.001)
Ever diagnosed with high blood pressure	-0.034*
	(0.001)
Ever diagnosed with lung disease	-0.054*
	(0.001)
Ever diagnosed with stroke	-0.067*
	(0.002)
IADL limitation only	-0.160*
	(0.002)
One or two ADL limitations	-0.099*
	(0.001)
Three or more ADL limitations	-0.149*
	(0.002)
Constant	0.881*
	(0.001)
N	19,676
Adjusted R^2	0.67

* $p < 0.01$

Table R: OLS regression of the predicted EQ-5D index score against chronic conditions and THEMIS-type functional status specification. p-values in parentheses. Data source: HRS, 1998. Sample included the age 51 and over community respondents. EQ-5D score was predicted using Model II in Table Q.

Functional status	Estimate	Predicted EQ-5D score	Age	Prevalence of chronic conditions: Cancer	Diabetes	Heart disease	Hypertension	Lung disease	Stroke
Healthy	Mean	0.841	63.9	0.114	0.142	0.189	0.481	0.072	0.045
	Sd	0.042	10.0	0.318	0.349	0.391	0.500	0.258	0.207
	Std. err	0.000	0.0	0.001	0.001	0.001	0.001	0.001	0.001
IADL limitation only - not in nursing home	Mean	0.655	70.0	0.151	0.223	0.334	0.581	0.152	0.162
	Sd	0.054	12.9	0.358	0.416	0.472	0.493	0.359	0.368
	Std. err	0.001	0.2	0.005	0.005	0.006	0.006	0.005	0.005
1 or 2 ADL limitations - not in nursing home	Mean	0.705	69.1	0.180	0.291	0.393	0.665	0.194	0.162
	Sd	0.057	12.0	0.384	0.454	0.488	0.472	0.395	0.368
	Std. err	0.000	0.1	0.003	0.003	0.004	0.003	0.003	0.003
3 or more ADL limitations - not in nursing home	Mean	0.636	70.9	0.181	0.361	0.467	0.691	0.216	0.314
	Sd	0.063	13.2	0.385	0.480	0.499	0.462	0.412	0.464
	Std. err	0.001	0.2	0.005	0.006	0.006	0.006	0.005	0.006
Nursing home residency	Mean	0.568	82.0	0.144	0.256	0.428	0.651	0.142	0.372
	Sd	0.077	10.0	0.351	0.437	0.495	0.477	0.349	0.484
	Std. err	0.001	0.2	0.006	0.008	0.009	0.008	0.006	0.009
All	Mean	0.808	65.2	0.125	0.170	0.229	0.514	0.094	0.076
	Sd	0.083	10.9	0.331	0.375	0.420	0.500	0.291	0.266
	Std. err	0.000	0.0	0.001	0.001	0.001	0.001	0.001	0.001

Table S: Average predicted EQ-5D score, age, and prevalence of chronic conditions by functional status for the stock THEMIS simulation sample (ages 51 and over in 2004). EQ-5D scores were predicted according to parameter estimates in Table R The predicted score for nursing home residents is reduced by 10% to account for the fact that the estimation sample in Table R only includes non-nursing home residents.

			Selection	1992	2010
Binary	working for pay		all	0.74	0.78
	non-zero wealth		all	0.97	0.98
	hypertension		all	0.30	0.40
	heart disease		all	0.09	0.10
	diabetes		all	0.07	0.13
	any health insurance		all	0.87	0.86
	SRH fair or poor		all	0.18	
Ordered	BMI status	normal	all	0.36	0.22
		overweight	all	0.40	0.35
		$30 \leq$ BMI < 35	all	0.17	0.25
		$35 \leq$ BMI < 40	all	0.04	0.09
		BMI ≥ 40	all	0.02	0.08
	Smoking status	never smoked	all	0.35	0.43
		former smoker	all	0.35	0.33
		current smoker	all	0.29	0.25
	Functional status	no ADL	all		
		no IADL	all		
Continuous	AIME (nominal $USD)		all		2,156.65
	quarters of coverage		all		100.54
Censored continuous	earnings		if working	45,722.90	47,105.12
	wealth		if non-zero	286,772.84	310,026.94
	DC wealth		if dc plan	18.63	25.29
Censored discrete	any DB plan		if working	0.44	0.44
	any DC plan		if working	0.25	0.26
Censored ordered	Early age eligible DB	<52		0.38	
		52-57		0.13	
		58>		0.36	
	Normal age eligible DB	<57		0.38	
		57-61		0.11	
		62-63		0.16	
		64>		0.10	
Covariates	hispanic		all	0.07	0.11
	black		all	0.10	0.12
	male		all	0.46	0.49
	less high school		all	0.21	0.10
	college		all	0.41	0.60
	single		all	0.19	0.27
	widowed		all	0.05	0.02
	cancer		all	0.04	0.06
	lunge disease		all	0.04	0.05

Table T: Initial conditions used for estimation (1992) and simulation (2010)

Covariate	Hypertension	Heart disease	Diabetes	Any health insurance	Self-reported health	Weight status	Smoking status	Functional status (ADL)	Functional status (IADL)	Working	Nonzero wealth	AIME	Quarters worked	IHT(HH wealth)	IHT(earned income)	Log(DC wealth)	Any DC plan	Any DB plan	Early retirement age	Normal retirement age
Non-Hispanic black	0.50	0.06	0.41	-0.08	0.49	0.38	-0.11	0.29	0.27	-0.02	-0.84	-19.32	0.23	-0.05	0.00	0.13	-0.10	-0.10		
Hispanic	0.02	-0.18	0.20	-0.67	0.48	0.17	-0.33	0.31	0.24	-0.12	-0.79	-18.11	-3.31	-0.04	-0.25	-0.23	0.01	0.01		
Less than high school	0.09	0.14	0.22	-0.52	0.48	0.12	0.29	0.24	0.40	-0.36	-0.40	-14.30	-2.99	-0.07	-0.29	-0.34	0.03	0.04		
Some college and above	-0.05	-0.06	-0.08	0.20	-0.38	-0.16	-0.12	-0.25	-0.35	0.29	0.88	14.20	5.21	0.11	0.25	0.20	-0.08	-0.14		
Male	0.07	0.29	0.08	-0.00	0.05	0.11	0.43	-0.07	-0.14	0.52	-0.01	-1.91	6.99	0.13	0.12	0.14	-0.08	0.00		
Single	0.17	0.04	0.05	-0.23	0.24	-0.04	0.31	0.10	0.02	0.08	-1.18	-26.16	0.43	-0.01	0.02	-0.04	0.01	0.04		
Widowed	0.21	0.07	0.28	-0.43	0.35	0.10	0.32	0.04	-0.05	-0.03	-1.24	-20.95	-0.30	-0.10	0.01	-0.11	-0.21	-0.23		
Lung disease	0.29	0.74	0.46	-0.10	1.11	0.09	0.59	0.83	0.23	-0.55	0.03	-18.29	-1.67	-0.02	-0.16	0.05	0.04	-0.02		
Cancer	0.10	0.21	0.09	0.25	0.71	-0.07	0.21	0.29	-0.01	-0.13	0.21	-1.01	0.56	-0.10	0.01	-0.03	-0.18	-0.27		
constant	-0.69	-1.56	-1.67	1.31	-1.21	0.29	0.09	-1.47	-1.32	0.44	2.78	71.13	15.26	0.76	-0.57	0.10	0.38	0.34		

Table U: Parameter estimates for latent model: conditional means and thresholds. Sample is respondents age 50-55 in 1992 HRS wave

	Hypertension	Heart disease	Diabetes	Any health insurance	Self-reported health	Weight status	Smoking status	Functional status (ADL)	Functional status (IADL)	Working	Nonzero wealth	AIME	Quarters worked	IHT(HH wealth)	IHT(earned income)	Log(DC wealth)	Any DC plan	Any DB plan	Early retirement age	Normal retirement age
Hypertension	1.000																			
Heart Disease	0.324	1.000																		
Diabetes	0.317	0.251	1.000																	
Any health insurance	-0.002	-0.008	-0.015	1.000																
Self-repoted health	0.334	0.480	0.423	-0.081	1.000															
Weight status	0.271	0.127	0.259	-0.004	0.164	1.000														
Smoking status	-0.010	0.051	0.037	-0.078	0.065	-0.088	1.000													
Functional status (ADL)	0.217	0.297	0.236	-0.069	0.506	0.143	0.031	1.000												
Functional status (IADL)	0.070	0.079	0.053	-0.025	0.148	-0.001	-0.033	0.210	1.000											
Working	-0.155	-0.280	-0.216	0.174	-0.419	-0.034	-0.044	-0.388	-0.170	1.000										
Nonzero wealth	-0.158	-0.192	-0.062	0.163	-0.135	-0.011	-0.044	-0.164	-0.178	0.339	1.000									
IHT(HH wealth)	-2.140	-2.346	-5.597	6.994	-6.560	-2.287	-4.666	-6.313	-2.414	2.601	0.000	1259.332								
IHT(earned income)	0.272	-0.060	-0.171	3.268	-1.359	0.026	-0.354	-1.114	-0.888	0.000	2.501	53.693	88.031							
Log(DC wealth)	0.011	-0.001	-0.001	0.032	-0.018	-0.020	-0.019	0.015	-0.016	0.000	0.001	2.711	0.973	0.085						
Any DC plan	0.029	0.056	0.058	0.477	-0.048	0.001	-0.054	-0.039	-0.033	0.000	0.123	2.817	3.490	0.000	1.000					
Any DB plan	0.043	0.056	0.019	0.667	-0.057	0.008	-0.079	-0.032	-0.022	0.000	0.148	1.479	4.447	0.025	0.733	1.000				
Early retirement age	0.086	-0.024	-0.068	0.122	0.003	0.012	-0.008	-0.043	0.022	0.000	-0.018	1.015	-0.212	0.021	-0.233	0.000	1.000			
Normal retirement age	0.081	-0.011	-0.019	0.116	-0.031	-0.003	0.010	-0.060	0.015	0.000	-0.040	0.197	-0.120	0.018	-0.207	0.000	0.835	1.000		

Table V: Parameter estimates for latent model: parameterized covariance matrix. Sample is respondents age 50-55 in 1992 HRS wave

	Ages 55-64			Ages 65 and over		
Payment sources	NHEA 2004 ($) (A)	THEMIS 2004, unadjusted ($) (B)	Adjustment factor (A)/(B)	NHEA 2010 ($) (C)	THEMIS 2010, unadjusted ($) (D)	Adjustment factor (C)/(D)
Total	7787.00	7117.00	1.09	18424.00	17266.00	1.07
Medicare	706.00	669.00	1.05	10016.00	9699.00	1.03
Medicaid	1026.00	665.00	1.54	2047.00	1152.00	1.78

Table W: Per capita medical spending by payment source, age group, and year

Year	2010	2030	2050
Population 51+ (Million)	100.11	133.87	157.32
Population 65+ (Million)	45.03	78.89	91.61
**Prevalence of selected conditions for ages 51+**			
Obesity (BMI >= 30) (%)	0.33	0.41	0.46
Overweight (25 <= BMI < 30) (%)	0.37	0.32	0.29
Ever-smoked	0.57	0.50	0.40
Smoking now	0.15	0.11	0.06
Diabetes	0.21	0.33	0.39
Heart disease	0.22	0.31	0.33
Hypertension	0.56	0.68	0.72
**Labor participation for ages 51+**			
Working (%)	0.44	0.37	0.36
Average earnings if working ($2010)	48089.62	51690.57	65916.22
**Government revenues from ages 51+ (Billion $2010)**			
Federal personal income taxes	438.23	575.17	948.70
Social security payroll taxes	120.56	167.08	257.27
Medicare payroll taxes	32.82	40.12	60.45
Total Revenue	591.60	782.37	1266.42
**Government expenditures from ages 51+ (Billion $2010)**			
Old Age and Survivors Insurance benefits (OASI)	667.89	1185.41	1701.88
Disability Insurance benefits (DI)	40.20	45.40	63.92
Supplementary Security Income (SSI)	21.48	28.14	41.73
Medicare costs	492.56	1336.36	2744.32
Medicaid costs	153.29	356.01	943.12
Medicare + Medicaid	1375.42	2951.33	5494.97
Total medical costs for ages 51+ (Billion $2010)	1175.01	3084.25	6409.60

Table X: Simulation results for status quo scenario

	"Obese 1980" Estimates		Relative Change from Status Quo		Absolute Change from Status Quo	
Year	2030	2050	2030	2050	2030	2050
Population 51+ (Million)	134.20	160.13	0.00	0.02	0.33	2.81
Population 65+ (Million)	79.07	94.16	0.00	0.03	0.18	2.55
**Prevalence of selected conditions for ages 51+**						
Obesity (BMI >= 30) (%)	0.31	0.26	-0.24	-0.44	-0.10	-0.20
Overweight (25 <= BMI < 30) (%)	0.35	0.35	0.08	0.19	0.03	0.05
Ever-smoked	0.50	0.40	0.00	0.01	0.00	0.00
Smoking now	0.11	0.06	-0.00	0.01	-0.00	0.00
Diabetes	0.28	0.28	-0.14	-0.28	-0.05	-0.11
Heart disease	0.30	0.32	-0.02	-0.05	-0.01	-0.02
Hypertension	0.67	0.69	-0.02	-0.05	-0.02	-0.03
**Labor participation for ages 51+**						
Working (%)	0.38	0.37	0.01	0.02	0.00	0.01
Average earnings if working ($2010)	51,856.61	66,026.53	0.00	0.00	166.04	110.31
**Government revenues from ages 51+ (Billion $2010)**						
Federal personal income taxes	584.51	986.19	0.02	0.04	9.34	37.49
Social security payroll taxes	169.48	266.49	0.01	0.04	2.40	9.22
Medicare payroll taxes	40.69	62.62	0.01	0.04	0.58	2.17
Total Revenue						
**Government expenditures from ages 51+ (Billion $2010)**						
Old Age and Survivors Insurance benefits (OASI)	1,186.38	1,742.27	0.00	0.02	0.98	40.39
Disability Insurance benefits (DI)	42.18	56.47	-0.07	-0.12	-3.22	-7.45
Supplementary Security Income (SSI)	28.23	40.36	0.00	-0.03	0.09	-1.38
Medicare costs	1,322.00	2,645.86	-0.01	-0.04	-14.36	-98.45
Medicaid costs	349.10	898.26	-0.02	-0.05	-6.91	-44.87
Medicare + Medicaid						
Total medical costs for ages 51+ (Billion $2010)	3,028.06	6,159.01	-0.02	-0.04	-56.19	-250.58

Table Y: Simulation results for obesity reduction scenario compared to status quo

Calendar year	National Wage Index	Real interest rate on wealth	COLA	Consumer Price Index	Substantial Gainful Activity	Y-o-Y excess real growth in medical costs
2004	35648.55	154.7553	3.606042	188.9	9720	.015
2005	36952.94	157.0766	3.703405	195.3	9960	.0148
2006	38651.41	158.3332	3.855245	201.6	10320	.0147
2007	40405.48	160.0749	3.982468	207.342	10800	.0145
2008	41334.97	163.1163	4.074064	215.303	11280	.0143
2009	42188.9	163.7688	4.31036	214.537	11760	.0141
2010	42907.15	171.4659	4.31036	214.537	12000	.0139
2011	43620.13	173.6949	4.31036	214.537	12000	.0138
2012	44197.64	176.4741	4.31036	214.537	12120	.0136
2013	44678.93	180.533	4.31036	214.537	12480	.0134
2014	45126.4	185.2268	4.31036	214.537	12840	.0133
2015	45737.88	190.7836	4.31036	214.537	13080	.0131
2016	46166.54	196.8887	4.31036	214.537	12699.34	.0129
2017	46633.77	202.5985	4.31036	214.537	12827.86	.0128
2018	47117.93	208.2712	4.31036	214.537	12961.05	.0126
2019	47609.11	214.1028	4.31036	214.537	13096.16	.0124
2020	48107.48	220.0977	4.31036	214.537	13233.25	.0122
2021	48615.77	226.2604	4.31036	214.537	13373.07	.0121
2022	49124.07	232.5957	4.31036	214.537	13512.89	.0119
2023	49625.12	239.1084	4.31036	214.537	13650.72	.0117
2024	50148.08	245.8035	4.31036	214.537	13794.57	.0115
2025	50681.91	252.6859	4.31036	214.537	13941.41	.0114
2026	51221.7	259.7611	4.31036	214.537	14089.9	.0112
2027	51773.64	267.0345	4.31036	214.537	14241.72	.011
2028	52331.86	274.5114	4.31036	214.537	14395.28	.0109
2029	52896.8	282.1978	4.31036	214.537	14550.68	.0107
2030	53472.1	290.0993	4.31036	214.537	14708.93	.0105
2031	54060.84	298.2221	4.31036	214.537	14870.88	.0104
2032	54659.91	306.5723	4.31036	214.537	15035.67	.0101
2033	55273.43	315.1563	4.31036	214.537	15204.43	.01
2034	55892.85	323.9807	4.31036	214.537	15374.82	.0097
2035	56518.25	333.0521	4.31036	214.537	15546.86	.0094
2036	57149.05	342.3776	4.31036	214.537	15720.37	.0091
2037	57790.2	351.9642	4.31036	214.537	15896.74	.0088
2038	58444.8	361.8192	4.31036	214.537	16076.81	.0085
2039	59104.39	371.9501	4.31036	214.537	16258.24	.0082
2040	59771.73	382.3647	4.31036	214.537	16441.81	.0079
2041	60445.75	393.0709	4.31036	214.537	16627.22	.0076
2042	61127.12	404.0769	4.31036	214.537	16814.65	.0073
2043	61816.84	415.3911	4.31036	214.537	17004.37	.007
2044	62511.55	427.022	4.31036	214.537	17195.47	.0067
2045	63211.19	438.9786	4.31036	214.537	17387.93	.0064
2046	63917.93	451.27	4.31036	214.537	17582.33	.0061
2047	64628.21	463.9056	4.31036	214.537	17777.72	.0058
2048	65348.03	476.895	4.31036	214.537	17975.72	.0055
2049	66072.87	490.248	4.31036	214.537	18175.11	.0052
2050	66803.52	503.9749	4.31036	214.537	18376.09	.0049

Table Z: Assumptions for each calendar year

Birth year	Normal Retirement Age	Delayed Retirement Credit
1920	780	.03
1921	780	.03
1922	780	.03
1923	780	.03
1924	780	.03
1925	780	.035
1926	780	.035
1927	780	.04
1928	780	.04
1929	780	.045
1930	780	.045
1931	780	.05
1932	780	.05
1933	780	.055
1934	780	.055
1935	780	.06
1936	780	.06
1937	780	.065
1938	782	.065
1939	784	.07
1940	786	.07
1941	788	.075
1942	790	.075
1943	792	.08
1944	792	.08
1945	792	.08
1946	792	.08
1947	792	.08
1948	792	.08
1949	792	.08
1950	792	.08
1951	792	.08
1952	792	.08
1953	792	.08
1954	792	.08
1955	794	.08
1956	796	.08
1957	798	.08
1958	800	.08
1959	802	.08
1960	804	.08

Table AA: Assumptions for each birth year. In years before 1920, all values are the same as in 1920. In years after 1960, all values are held constant at their 1960 levels.

References

[1] Atherly, A., Dowd, B. E., and Feldman, R. (2004). The effect of benefits, premiums, and health risk on health plan choice in the medicare program. *Health services research*, 39(4p1):847–864.

[2] Bhattacharya, J., Shang, B., and Goldman, D. P. (2005). Technological advances in cancer and future spending by the elderly. *Health Affairs*, 24(Suppl 2):W5R53–66.

[3] Brazier, J. E. and Roberts, J. (2004). The estimation of a preference-based measure of health from the sf-12. *Medical care*, 42(9):851–859.

[4] Buchmueller, T. (2006). Price and the health plan choices of retirees. *Journal of Health Economics*, 25(1):81–101.

[5] Council, N. R. (2010). *Improving health care cost projections for the Medicare population: Summary of a workshop*. The National Academies Press.

[6] Department of Health and Human Services, National Committee on Vital and Health Statistics, Subcommittee on Population Health (2009). Transcript of the February 27, 2009 meeting.

[7] Dolan, P. (1997). Modeling valuations for euroqol health states. *Medical care*, 35(11):1095–1108.

[8] Fryback, D. G., Dunham, N. C., Palta, M., Hanmer, J., Buechner, J., Cherepanov, D., Herrington, S., Hays, R. D., Kaplan, R. M., Ganiats, T. G., et al. (2007). Us norms for six generic health-related quality-of-life indexes from the national health measurement study. *Medical care*, 45(12):1162–1170.

[9] Goldman, D. P., Cutler, D. M., Shang, B., Bhattacharya, J., Joyce, G. F., Lakdawalla, D. N., Panis, C., and Shekelle, P. G. (2005). Consequences of health trends and medical innovation for the future elderly. *Health Affairs*, 24(Suppl 2):W5R5–17.

[10] Goldman, D. P., Cutler, D. M., Shang, B., and F, J. G. (2006). The value of elderly disease prevention. *Forum for Health Economics & Policy*, 9(2):28.

[11] Goldman, D. P., Michaud, P. C., Lakdawalla, D. N., Zheng, Y., Gailey, A., and Vaynman, I. (2010). The fiscal consequences of trends in population health. *National Tax Journal*, 63(2):307–330.

[12] Goldman, D. P., Shekelle, P. G., Bhattacharya, J., Hurd, M., and Joyce, G. F. (2004). Health status and medical treatment of the future elderly. Technical report, DTIC Document.

[13] Goldman, D. P., Zheng, Y., Girosi, F., Michaud, P. C., Olshansky, S. J., Cutler, D. M., and Row, J. W. (2009). The benefits of risk factor prevention in americans aged 51 years and older. *American Journal of Public Health*, 99(11):2096–2101.

[14] Honeycutt, A. A., Boyle, J. P., Broglio, K. R., Thompson, T. J., Hoerger, T. J., Geiss, L. S., and Narayan, K. V. (2003). A dynamic markov model for forecasting diabetes prevalence in the united states through 2050. *Health care management science*, 6(3):155–164.

[15] Lakdawalla, D. N., Goldman, D. P., Michaud, P. C., Sood, N., Lempert, R., Cong, Z., de Vries, H., and Gutierrez, I. (2009). Us pharmaceutical policy in a global marketplace. *Health Affairs*, 28(1):w138–150.

[16] Lakdawalla, D. N., Goldman, D. P., and Shang, B. (2005). The health and cost consequences of obesity among the future elderly. *Health Affairs*, 24(2):W5R30–41.

[17] Levy, D. (2006). Trends in smoking rates under different tobacco control policies: results from the SimSmoke tobacco policy simulation model.

[18] Lubitz, J. (2005). Health, technology, and medical care spending. *Health Affairs*, 24(2):W5R81–5.

[19] MacKinnon, J. G. and Magee, L. (1990). Transforming the dependent variable in regression models. *International Economic Review*, pages 315–339.

[20] Mainous III, A., Baker, R., Koopman, R., Saxena, S., Diaz, V., Everett, C., and Majeed, A. (2007). Impact of the population at risk of diabetes on projections of diabetes burden in the united states: an epidemic on the way. *Diabetologia*, 50(5):934–940.

[21] Michaud, P. C., Goldman, D. P., Lakdawalla, D. N., Zheng, Y., and Gailey, A. H. (2012). The value of medical and pharmaceutical interventions for reducing obesity. *Journal of Health Economics*, 31(4):630–643.

[OECD] OECD. Taxing wages 2004.

[22] Office, C. B. (2012). Raising the excise tax on cigarettes: Effects on health and federal budget. Technical report.

[23] Poterba, J., Venti, S., and Wise, D. A. (2007). The decline of defined benefit retirement plans and asset flows. Technical report, National Bureau of Economic Research.

[24] Poterba, J. M., Venti, S. F., and Wise, D. A. (2008). New estimates of the future path of 401 (k) assets. In *Tax Policy and the Economy, Volume 22*, pages 43–80. University of Chicago Press.

[25] Poterba, J. M., Venti, S. F., and Wise, D. A. (2009). The decline of defined benefit retirement plans and asset flows. In *Social Security policy in a changing environment*, pages 333–379. University of Chicago Press.

[26] Roodman, D. (2011). Fitting fully observed recursive mixed-process models with cmp. *Stata Journal*, 11(2):159–206(48).

[27] Ruhm, C. J. (2007). Current and future prevalence of obesity and severe obesity in the united states. In *Forum for Health Economics & Policy*, volume 10.

[28] Selden, T. M. and Sing, M. (2008). Aligning the Medical Expenditure Panel Survey to aggregate us benchmarks. Technical report, Agency for Healthcare Research and Quality.

[29] Shaw, J. W., Johnson, J. A., and Coons, S. J. (2005). Us valuation of the eq-5d health states: development and testing of the d1 valuation model. *Medical care*, 43(3):203–220.

[30] THE BOARD OF TRUSTEES, FEDERAL OLD-AGE AND SURVIVORS INSURANCE AND FEDERAL DISABILITY INSURANCE TRUST FUNDS (2009). The 2009 annual report of the board of trustees of the federal old-age and survivors insurance and federal disability insurance trust funds. Technical report, Washington, D.C.

[31] THE BOARDS OF TRUSTEES, FEDERAL HOSPITAL INSURANCE AND FEDERAL SUPPLEMENTARY MEDICAL INSURANCE TRUST FUNDS (2007). 2007 annual report of the boards of trustees of the federal hospital insurance and federal supplementary medical insurance trust funds. Technical report, Washington, D.C.

[32] U.S. Census Bureau (2012). U.s. census bureau projections show a slower growing, older, more diverse nation a half century from now. access on Jan 27, 2017.

[33] US Senate Committee on Health, Education, Labor and Pensions (2008). Examining disease prevention and public health, focusing on transforming the health care system.

[34] Wooldridge, J. M. (2000). A framework for estimating dynamic, unobserved effects panel data models with possible feedback to future explanatory variables. *Economics Letters*, 68(3):245–250.
