# Supplementary material for: Lifetime benefits of early detection and treatment of diabetic kidney disease
Source: PLoS One. 2019 May 31;14(5):e0217487. doi: 10.1371/journal.pone.0217487 (PMC6544227; doi:10.1371/journal.pone.0217487)
Supplement: S2 Appendix — (DOCX) [file pone.0217487.s002.docx]

Lifetime benefits of early detection and treatment of diabetic kidney disease

**Technical Appendix 2: Implementation of THEMIS for this study**

Table of Contents

[Study design 3](#_Toc496284670)

[Study population 3](#_Toc496284671)

[Data sources for the DKD module 3](#_Toc496284672)

[Description of lab values in NHANES 3](#_Toc496284673)

[Imputing DKD using the NHANES model in the HRS population 4](#_Toc496284674)

[Key study variables 4](#_Toc496284675)

[Outcomes/endpoint variables 4](#_Toc496284676)

[Exposure/Independent Variables of Interest 5](#_Toc496284677)

[Other covariates/control variables 5](#_Toc496284678)

[Statistical analysis 6](#_Toc496284679)

[Identifying patients with DKD in NHANES 6](#_Toc496284680)

[Imputation of DKD status into the HRS 7](#_Toc496284681)

[Incidence model development 8](#_Toc496284682)

[DKD incidence 8](#_Toc496284683)

[Any stage 5 CKD incidence 9](#_Toc496284684)

[Diagnosis of DKD or stage 5 CKD 9](#_Toc496284685)

[Calibration of DKD and stage 5 CKD-related outcomes 10](#_Toc496284686)

[Scenario simulations 11](#_Toc496284687)

[Simulating the effects of a novel DKD biomarker on outcomes associated with DKD in THEMIS 11](#_Toc496284688)

[Additional scenarios: Combining the biomarker with more effective treatment 12](#_Toc496284689)

[Discounting 13](#_Toc496284690)

[Costs 13](#_Toc496284691)

[Modeling cycle 13](#_Toc496284692)

[Results stratification 13](#_Toc496284693)

[References 13](#_Toc496284694)

# Study design

This study used THEMIS to model health and economic outcomes for individuals with diabetic kidney disease (DKD). To do so, a module was added to THEMIS to identify individuals with DKD, determine the diagnosis status of each patient, and measure their health and economic outcomes. This technical appendix (Appendix 2) provides detail on the application of THEMIS to this study. Additional detail on THEMIS is available in a separate technical appendix (Appendix 1).

In addition to the base case scenario, our implementation explored one main scenario that deviated from the status quo: the implementation of a biomarker test that identifies individuals at risk of developing DKD or DKD patients at risk of progressing rapidly. Two additional scenarios were also considered: the biomarker combined with a 50% more effective treatment, and the biomarker combined with a 100% more effective treatment.

# Study population

THEMIS tracks individuals over age 50 throughout their remaining lives to project their disease and comorbidity burdens, life expectancy, functional status, health care costs, employment status, and transfer payments (including disability insurance receipt) up until 2050 using data from the Health and Retirement study. The HRS is a biennial, nationally representative, longitudinal study of Americans over age 50. A number of publications have used THEMIS to project the consequences of disability trends for the US.^1-5^ Others have used THEMIS to estimate the benefits of diabetes prevention.^6,7^

In this study, we built a DKD THEMIS module using data from the National Health and Nutrition Survey (NHANES). The planned development of that module, including the algorithm for identifying individuals with or at risk of developing DKD, is described in the statistical analysis section below.

# Data sources for the DKD module

As explained in greater detail in the statistical analysis section below, we built a module to impute DKD status in THEMIS. Two data sources were required to build this module:

- National Health And Nutritional Examination Survey
- Health and Retirement Study

The cohorts in THEMIS are based on data from the HRS. However, as the HRS does not contain biomarker data, NHANES was used to impute DKD status into THEMIS. status based on survey questions (e.g., has a doctor ever told you that you have diabetes, has a doctor ever told you that you have kidney disease) and laboratory values (i.e., albuminuria and serum creatinine levels), to develop an algorithm to impute DKD status based on observed demographic characteristics common to the NHANES and the HRS. Although NHANES is well suited to the identification of disease (both diagnosed and undiagnosed) due to its inclusion of laboratory values, it is of limited use for incidence and progression calculations due to its cross-sectional nature. An ideal source would include laboratory values in a longitudinal dataset that is representative of the US population.

### Description of lab values in NHANES

We used the variables listed below to calculate the albumin/creatinine ratio (ACR) and the estimated glomerular filtration rate (eGFR) measures to determine DKD status in NHANES. (For details, please see the statistical analysis section.)

- URXUMA- Albumin, urine (µg/mL)
- URXUCR- Creatinine, urine mg/dL
- LBXSCR - Creatinine (mg/dL)

### Imputing DKD using the NHANES model in the HRS population

Once DKD status, whether diagnosed or undiagnosed, was identified in NHANES, it was imputed for individuals in the HRS as a function of demographic variables, health status, and risk factors which are common to both datasets.^8^ The imputation methodology is further detailed in the statistical analysis section. There were some limitations to this imputation method. First, NHANES data is not longitudinal, which makes modeling incidence of conditions impractical. Second, there may be factors important to our outcomes that are not observed in the target dataset, such as detailed clinical and laboratory data. Third, correlations with other outcomes (i.e., variables not used as part of the imputation) could not be preserved because we could not include all possible outcomes and still achieve convergence and a good fit to the target dataset.

# Key study variables

## Outcomes/endpoint variables

The model considered the following outcomes of interest:

- *Life expectancy* was measured as the average life expectancy of the population of interest conditional on surviving past age 50.
- *Quality of life* was computed based on the EuroQol five dimensions questionnaire (EQ-5D-3L). As the HRS does not ask the appropriate questions to impute EQ-5D-3L and the MEPS does, we imputed EQ-5D-3L scores into the HRS. More information on the imputed EQ-5D-3L model can be found in the Appendix 1 on the THEMIS model.
- *Medical expenses* were inflated to 2015 dollars in real terms according to the CBO projections, which are tied to the real growth in GDP.^9^
  - *Total medical spending* was defined as the total medical expenditures (all inclusive, regardless of payer) for the patients involved.
  - *Medicare spending* was defined as the total Medicare expenditures (Parts A, B, and D) for the patients involved. THEMIS does not explicitly model Medicare health maintenance organization (HMO) plans, but instead assumes everyone participates in FFS plans.
  - *Medicaid spending* was measured by the total Medicaid expenditures for the patients involved.
- *Other government spending* included disability insurance, Social Security retirement benefits, and Supplemental Security Income (SSI). All dollar figures are reported in 2015 USD.
  - *Disability insurance* participation was modeled for the federal Social Security Disability Insurance (SSDI) program as a binary outcome (probit) and the benefit amount was computed algorithmically. Details on the modeling of disability insurance can be found in Appendix 1 on the THEMIS model.
  - *Social Security retirement benefits* were modeled at the individual and family level, meaning that spouse and widow payments are properly accounted for. The claiming decision was modeled first, including the time-variant normal retirement age, followed by the benefit computation. In the case of individuals retiring either before or after their normal retirement age, the benefits were adjusted appropriately (reduced for early retirement, increased for later retirement).
  - *Supplemental Security Income* (SSI) was modeled at the individual level and is a prediction of program participation and an algorithmic calculation of benefits received by each individual claiming SSI.

## Exposure/Independent Variables of Interest

The key variable of interest is DKD status. Specifically, we compared outcomes of individuals with diabetes and CKD to those of individuals with diabetes but no CKD.^[[1]](#footnote-1)^ CKD has been associated with premature mortality.^10^

Diabetes was defined by the survey question: “Has a doctor ever told you that you have diabetes?” Only patients with diagnosed diabetes were considered.

Among individuals with DKD, we differentiated between those who have stage 5 CKD and those who do not. Stage 5 CKD is treated by hemodialysis, peritoneal dialysis, or by having a kidney transplant.^11^ Stage 5 CKD has large effects on an individual’s productivity, especially for patients on dialysis.^12-14^ Dialysis is paid for by Medicare under the Medicare stage 5 CKD Reimbursement program, the Social Security Amendments of 1972 (Public Law 92-603), and the stage 5 CKD Program Amendments of 1978 (Public Law 95-292). Hence, progressing to stage 5 CKD has large financial consequences for the federal government and the larger American society.^15,16^

Laboratory values were used to assign DKD status to individuals in the NHANES. Those who qualified as having DKD were divided into two categories (diagnosed and undiagnosed) by whether or not the respondent reported being told about kidney disease by a doctor. Patients who responded that a doctor had diagnosed them with DKD were flagged as diagnosed DKD patients regardless of their lab values.

## Other covariates/control variables

To implement the DKD module within THEMIS, the specification of the model of DKD status could only include variables both available in the HRS and NHANES as controls. These included the following:

- Age: measured in brackets (50-54, 55-59, 60-64, 65-69, 70-74, 75-79, 80 and over)^17,18^
- Gender: male or female^17,18^
- Race/ethnicity: black non-Hispanic, Hispanic, or other non-Hispanic ^17^
- Marital status: married, widowed, or other
- Educational attainment: less than high school, high school graduate, or some college and above
- Smoking status: current smoker, ex-smoker, never smoked^17,18^
- BMI: defined as:^19^
  - Underweight: [0,18.5) kg/m^2^
  - Normal: [18.5, 25) kg/m^2^
  - Overweight: [25, 30) kg/m^2^
  - Obese I (moderately obese): [30, 35) kg/m^2^
  - Obese II (severely obese): [35, 40) kg/m^2^
  - Obese III (very severely obese): [40, ∞) kg/m^2^
- Comorbid conditions, defined by survey questions “Has a doctor ever told you that you have …?”:
  - Cancer
  - High blood pressure (which is a measure for hypertension)^17,18^
  - Heart disease
  - Lung disease
  - Stroke

# Statistical analysis

## Identifying patients with DKD in NHANES

We first identified patients with DKD in the NHANES dataset using a combination of survey questions and laboratory values. This approach enabled us to capture patients with DKD who may or may not have been diagnosed. Specifically, we looked at survey questions that ask about a prior diagnosis diabetes and kidney disease (e.g., “Other than pregnancy, have you ever been told by a doctor or health professional that you have diabetes or sugar diabetes?”, “Have you ever been told by a doctor or other health professional that you had weak or failing kidneys? Do not include kidney stones, bladder infections, or incontinence.”). We focused on the type 2 diabetes population by excluding any patients who answered with any age prior to age 16 to the survey question “How old were you when a doctor or other health professional first told you that you had diabetes or sugar diabetes?” as this is a proxy for type 1 diabetes (or juvenile diabetes). We note that some individuals with type 1 diabetes are diagnosed after age 16, and some individuals with type 2 diabetes may be diagnosed in childhood, so this method of separating type 1 diabetes from type 2 diabetes is approximate.

To determine whether individuals had DKD regardless of diagnosis status, we used laboratory values recorded for albuminuria and serum creatinine levels to determine CKD status.

DKD was defined as diabetes with albuminuria (ACR ≥30 mg/g), impaired eGFR (<60 mL/min/1.73 m^2^), or both.

Following Coresh et al. (2007), we captured patients with both microalbuminuria (ACR >30 mg/g and ≤299 mg/g) and clinical albuminuria (ACR ≥300 mg/g).^20^ The ACR was calculated as:^21^

$ACR \left( mg/g \right)=\frac{Urinary albumin (mg/dl)}{Urinary creatinine (mg/dl)}\times1000$.

We obtained the eGFR using serum creatinine lab values and age, sex, race, and BMI. The National Kidney Foundation recommends the use of the CKD-EPI Creatinine Equation (2009) to estimate GFR.^22^ The CDK-EPI Creatinine Equation was calculated as follows:^23^

$$eGFR=141\times{\min(\frac{S_{Cr}}{\kappa},1)}^{\alpha}\times{max(\frac{S_{Cr}}{\kappa},1)}^{-1.209}\times{0.993}^{Age}\times1.018 \left( if female \right)\times1.159(if black)$$

$eGFR=mL/min/1.73m^{2}$

$$S_{Cr}=mg/dL$$

$$\kappa=0.7 \left( females \right) or 0.9 (males)$$

$$\alpha=-0.392 \left( females \right) or-0.411 (males)$$

Note that the above methods enabled us to identify both DKD and CKD (i.e., patients with CKD but not necessarily diabetes). Although the focus of this study is DKD, we also tracked CKD status to be able to compare our algorithm estimates of CKD prevalence in the literature as a validation exercise. Note also that the use of creatinine based equations to estimate GFR is imperfect, particularly for those with unstable renal function or extremes in muscle mass and diet. Estimates are less accurate in these populations.^24^

We then compared the overlap across the survey questions and lab values. If a patient is identified as having DKD according to their answers to survey questions but not according to their lab values, we still counted them as having DKD. (Lab values can change with treatment.)

Individuals were classified as having diagnosed DKD with stage 5 CKD when they replied yes to the questions: “Other than pregnancy, have you ever been told by a doctor or health professional that you have diabetes or sugar diabetes?”’ and “Has a doctor ever told you that you have kidney disease requiring renal dialysis?” The latter question was only available in waves 1999-2000, 2001-2002, 2003-2004 of the NHANES. Using the common CKD severity scale, we labeled undiagnosed stage 5 CKD among those patients we identified as having DKD, based on eGFR values (eGFR <15 mL/min/1.73 m^2^).^20^ Stage 5 CKD is much costlier and has greater productivity implications, including from premature mortality, than earlier stages of DKD. Therefore, we classified DKD severity as non-stage 5 CKD and stage 5 CKD.

Using the NHANES population, we determined the distribution of patient health and demographic characteristics within the DKD population. In particular, all baseline characteristics that enter into THEMIS – e.g., disease prevalence, age, gender, smoking status, race/ethnicity, educational attainment, marital status, body mass index – were measured for the DKD population.

We also generated distributions of CKD and DKD by stage 5 CKD status, and tabulated these according to whether patients have been diagnosed with kidney disease.

Descriptive statistics of the NHANES population are reported in Table A.

**Table A: Descriptive statistics of the diabetes and DKD populations in NHANES**

| **Variable** | **Diabetes** | **DKD** | **Diagnosed DKD** | **Lab-based DKD** | **Stage 5 DKD** | **Diagnosed Stage 5 DKD** | **Lab-based Stage 5 DKD** |
| --- | --- | --- | --- | --- | --- | --- | --- |
| N (Unweighted) | 3,806 | 1,776 | 361 | 1,681 | 50 | 11 | 44 |
| Age (mean, SD) | 65.75 (9.34) | 69.00 (9.24) | 67.13 (8.68) | 69.16 (9.22) | 64.31 (9.23) | 62.40 (9.93) | 63.52 (9.51) |
| Male | 48% | 47% | 46% | 48% | 49% | 50% | 44% |
| Race/Ethnicity |  |  |  |  |  |  |  |
| Black, non-Hispanic | 16% | 17% | 22% | 17% | 48% | 18% | 54% |
| Hispanic | 11% | 10% | 10% | 10% | 17% | 11% | 17% |
| BMI |  |  |  |  |  |  |  |
| Obese | 50% | 52% | 54% | 52% | 29% | 31% | 25% |
| Underweight | 0% | 0% | 1% | 0% | 4% | 0% | 5% |
| Overweight | 32% | 29% | 29% | 29% | 36% | 48% | 39% |
| Normal weight | 17% | 19% | 17% | 19% | 31% | 21% | 32% |
| Smoking behavior |  |  |  |  |  |  |  |
| Current smoker | 14% | 14% | 14% | 14% | 11% | 0% | 12% |
| Ever smoked | 39% | 41% | 43% | 41% | 33% | 32% | 31% |
| Comorbidities |  |  |  |  |  |  |  |
| Hypertension | 71% | 78% | 83% | 78% | 91% | 80% | 97% |
| Heart Disease | 28% | 38% | 49% | 38% | 70% | 85% | 68% |
| Stroke | 11% | 16% | 23% | 16% | 23% | 12% | 26% |
| Cancer | 20% | 21% | 21% | 21% | 5% | 6% | 6% |
| Lung | 5% | 6% | 9% | 6% | 9% | 31% | 2% |
| Education |  |  |  |  |  |  |  |
| Less than high school | 32% | 38% | 44% | 37% | 44% | 24% | 47% |
| High school graduate/GED | 26% | 27% | 21% | 27% | 14% | 24% | 8% |
| Some college or AA degree | 24% | 23% | 26% | 23% | 28% | 52% | 29% |
| College graduate or above | 17% | 13% | 9% | 13% | 13% | 0% | 15% |
| Marital status |  |  |  |  |  |  |  |
| Married | 60% | 54% | 56% | 54% | 54% | 85% | 49% |
| Widowed | 18% | 25% | 21% | 24% | 19% | 6% | 22% |
| Blood pressure |  |  |  |  |  |  |  |
| SBP (mean, SD) | 132.46 (20.60) | 137.13 (22.89) | 135.51 (26.46) | 137.26 (22.93) | 136.79 (30.74) | 127.75 (28.13) | 136.07 (31.52) |
| DBP (mean, SD) | 66.42 (14.71) | 64.51 (16.59) | 64.01 (16.85) | 64.25 (16.66) | 58.92 (17.81) | 49.39 (19.88) | 57.87 (18.95) |

##

## Imputation of DKD status into the HRS

Using the previously defined DKD measure from the NHANES, we developed a model to predict the likelihood of DKD as well as the severity of DKD. Specifically, the model classified individuals with diabetes into five groups: non-DKD, undiagnosed DKD without stage 5 CKD, undiagnosed DKD with stage 5 CKD, diagnosed DKD without stage 5 CKD, and diagnosed DKD with stage 5 CKD. The model was estimated as a two-stage probit (1: DKD versus no DKD; 2: among DKD, stage 5 CKD versus no stage 5 CKD), and an independent probit for diagnosis status that includes an indicator for stage 5 CKD. Model selection was determined by goodness of fit. The model was estimated in NHANES using parameters common to the HRS. Seven different specifications were estimated to achieve the highest goodness of fit, including:

1. age (continuous) and indicators for male, smokes now, black, Hispanic, underweight, overweight, obese, and widowed;
2. age (continuous) and indicators for male, smokes now, black, Hispanic, underweight, overweight, obese I, obese II, obese III, and widowed;
3. indicators for ages 50-59, ages 60-69, ages 70-79, ages 80 and over, smokes never, male, black, Hispanic, underweight, overweight, obese I, obese II, obese III, and widowed;
4. Same covariates as (3) plus an indicator for married;
5. Same covariates as (4) plus indicators for education (having less than high school, and having some college or more);
6. Same covariates as (5) plus an indicator for smokes ever; and
7. Same covariates as (6) plus indicators for ever having hypertension, heart disease, stroke, cancer, and lung disease.

The specification with the highest goodness of fit was specification 7, which was used for the model.

We then predicted the prevalence of DKD in the diabetic HRS population using the above NHANES model. Additionally, we assigned CKD severity (i.e. non-stage 5 CKD vs. stage 5 CKD) in the HRS population using the same model.

In patients with a DKD-related diagnosis, we assumed this is an absorbing state (i.e., once patient is diagnosed with DKD, they cannot become undiagnosed with DKD in later years). Patients predicted to have undiagnosed DKD could transition to diagnosed DKD in future time periods or remain in the undiagnosed state for the course of their lifetime.

Once we had estimated the size of the DKD population in the HRS, we compared the distribution to the distribution of the NHANES population with DKD as a method of validation.

## Incidence model development

### DKD incidence

To compute an incidence rate for DKD, we used the incidence rates by age group from the ‘Analysis of direct costs associated with DKD’-study as a basis.^25^ This study used the 2011-2013 Truven MarketScan data to identify patients with diabetes and DKD using ICD-9 codes for diabetes and CKD. The incidence rates are reported in Table B.

Table B: Incidence rates from ‘Analysis of direct costs associated with DKD’ study

| Population | Incidence rate (cases per 1,000 patient-years) | Derived 2-year incidence rate |
| --- | --- | --- |
| Ages 18-64 | 23.4 | 0.0046 |
| Ages 65 and up | 69.3 | 0.0138 |

We used a logistic regression as our transition function, such that the probability of transitioning to DKD status can be written as:

$$\mathbb{P}\left( DKD | diabetes \right)=\frac{1}{1+e^{-\beta_{1}x-\beta_{0}}}$$

where $x$ is 0 when the respondent is under age 65, and 1 when the respondent is age 65 or over.

The logistic coefficients are derived from two equations and two unknowns:

$$\frac{1}{1+e^{-\beta_{0}}}=0.0046$$

$$\frac{1}{1+e^{-\beta_{1}x-\beta_{0}}}=0.0138$$

Solving for these two equations gives $\beta_{0}=-5.377$ and $\beta_{1}=1.108$. Thus, the probability of DKD given the respondent has diabetes and not yet diagnosed is:

$$\mathbb{P}\left( DKD | diabetes \right)=\frac{1}{1+e^{-1.108x+5.377}}$$

### Any stage 5 CKD incidence

According to the National Institute of Diabetes and Digestive Kidney Disease, the stage 5 CKD incidence rate is roughly 1,000 per million for African Americans and 375 cases per million for all Americans.^26^ The prevalence of African Americans in the THEMIS simulation cohort (aged 51+ in 2010) is 9.8%. Thus, the stage 5 CKD incidence rate for non-African Americans (i.e., other) can be derived from the following equation:

$$375=1,000*0.098+P_{other}*(1-0.098)$$

$P_{other}$ is then approximately 305 cases per million.

The probability of transitioning to stage 5 CKD status can be written as:

$$\mathbb{P}\left( stage 5 CKD | diabetes \right)=\frac{1}{1+e^{-\beta_{1}x-\beta_{0}}}$$

where $x$ is 0 when the respondent is non-African American, and 1 when the respondent is African American.

Again, the logistic coefficients are derived from two equations and two unknowns:

$$\frac{1}{1+e^{-\beta_{0}}}=\frac{305}{1,000,000}$$

$$\frac{1}{1+e^{-\beta_{1}x-\beta_{0}}}=\frac{1,000}{1,000,000}$$

Solving for these two equations gives $\beta_{0}=-8.09$ and $\beta_{1}=1.18$. Thus, the probability of stage 5 CKD given the respondent is diabetic and not yet diagnosed is:

$$\mathbb{P}\left( stage 5 CKD | diabetes \right)=\frac{1}{1+e^{-1.18x+8.09}}$$

### Diagnosis of DKD or stage 5 CKD

As the data for this study does not include incidence data for diagnosis, the incidence rate was calculated from the following equation:

$$P_{t+1}=P_{t}+I_{t}-D_{t}$$

where $P$ is the number of people with the disease, $I$ is the number of new cases, and $D$ is the number of people with the disease who have died.

The two-year mortality rate in the THEMIS data is 7.9% for patients with diabetes. Table C shows the hazard ratios for patients with diabetes at various stages of kidney disease reported by a study by Fox et al. (2012) and the resulting two-year mortality rates.^27^

Table C: All-cause mortality in patients with diabetes

| Stage | Hazard ratio (to normal) | Two-year mortality |
| --- | --- | --- |
| DKD, no stage 5 CKD | 1.18-2.28 | 9.32% - 18.00% |
| Stage 5 CKD | 4.46 | 32.2% |

Using the numbers of Table C in the incidence equation, the incidence of diagnosis can be calculated using the following two equations (taking the midpoint of the range for DKD, non-stage 5 CKD, i.e. 13.7%):

$$P_{t+1,DKD}=P_{t,DKD}(1-0.137)+I_{t,DKD}$$

$$P_{t+1,stage 5 CKD}=P_{t,stage 5 CKD}(1-0.322)+I_{t,stage 5 CKD}$$

where $D=MP$, with $M$ as the mortality rate. Using the prevalence estimates over time from the NHANES, $I$ can be found through the least squares method, assuming the incidence rate is not time-varying over the analysis period. The regression results show that $I_{DKD}=0.9\%$ and $I_{ESRD}=0.2\%$.

The probability of transitioning to can be written as:

$$\mathbb{P}\left( DKD | diabetes \right)=\frac{1}{1+e^{-\beta_{1}x-\beta_{0}}}$$

where $x$ is 0 when the respondent does not have stage 5 CKD, and 1 when the respondent has stage 5 CKD.

The logistic coefficients are derived from two equations and two unknowns:

$$\frac{1}{1+e^{-\beta_{0}}}=0.009$$

$$\frac{1}{1+e^{-\beta_{1}x-\beta_{0}}}=0.002$$

Solving for these two equations gives $\beta_{0}=-4.70$ and $\beta_{1}=-1.51$. Thus, the probability of DKD given the respondent is diabetic and not yet diagnosed is:

$$\mathbb{P}\left( DKD | diabetes \right)=\frac{1}{1+e^{1.51x+4.70}}$$

## Calibration of DKD and stage 5 CKD-related outcomes

Parameters from the literature were used to estimate how DKD affects outcomes—namely mortality, utility, healthcare costs, and productivity. The relative risk of mortality in individuals with DKD is 2.68 compared to individuals with diabetes and no kidney disease^[[2]](#footnote-2)^.^28^ In addition, the hazard ratio of all-cause mortality is 4.46 in individuals with diabetes and stage 5 CKD compared to those with diabetes and no kidney disease^[[3]](#footnote-3)^.^27^ Based on a clinical trial population-based study, renal failure reduces utility by 0.049 in people with diabetes. ^29^

In terms of costs, the US renal data system annual report from 2015 estimates that healthcare costs are 59% higher for patients with non-stage 5 DKD compared to costs of patients with diabetes but no kidney disease^[[4]](#footnote-4)^.^30^ Furthermore, healthcare costs are 362% higher for patients with diabetes and stage 5 CKD compared to costs of patients with diabetes but no kidney disease^[[5]](#footnote-5)^.^30^ (However, these increased costs are mitigated to some extent by the fact that people with diabetes and stage 5 CKD have higher mortality rates than patients with diabetes but no kidney disease.) In terms of productivity, individuals with stage 5 CKD are 7.17 times more likely not to be working than the general population whereas they are no more or less likely to be working than people with stage 5 CKD and no diabetes^[[6]](#footnote-6)^.^31^

Since the DKD states are not native to the HRS, MCBS, or MEPS (the sources for all the above modeled outcomes), the models were calibrated to hit the literature results as closely as possible. For instance, after adjusting for the higher mortality rate listed above, probability of diagnosis for stage 5 CKD patients was increased by 30% to match the diagnosis rates observed in the NHANES.

## Scenario simulations

### Simulating the effects of a novel DKD biomarker on outcomes associated with DKD in THEMIS

We ran a scenario where we assumed there is implementation of a novel biomarker that can identify patients at high risk of developing DKD or DKD patients at risk of progressing rapidly. Under this scenario, we allowed patients to vary in terms of when/whether they are treated for DKD. Some should be treated but will not be. Others will be treated later in the course of their disease. Still others will be treated earlier due to the biomarker.

Specifically, we assume 30% of patients tested, chosen at random each year, return positive for the biomarker, putting them at higher risk for DKD. We compared results in medical expenditures, QALYs, survival gains, employment, disability, and earnings to a status quo scenario in which we assume the biomarker is not implemented.

To model this scenario, we assumed the following.

1. Everyone with diabetes is tested for the biomarker every 2 years as a hypothetical example.
2. The test costs $35 including administration costs.^[[7]](#footnote-7)^
3. The biomarker identifies the following:
   1. Individuals with DKD that is not yet detected/diagnosed or at risk of DKD
      - 30% of tested individuals will have a positive biomarker test result
      - For individuals with a positive biomarker result, the probability of progressing to DKD is 2.5 times greater than that of those with a negative result^32^
      - In base case, we use a logit model to determine the probability that DKD is diagnosed. Using the biomarker, we assume that the rate of DKD diagnosis increases to 2X because at-risk individuals are being more closely monitored
   2. Individuals with diagnosed DKD who are at risk of rapid progression
      - 30% of tested individuals will have a positive result
      - For individuals with a positive biomarker result, the probability of progressing is 2.5 times greater than that of those with a negative result.^32^
4. When people are identified by the biomarker,
   1. Those diagnosed with DKD (including the additional people diagnosed due to the use of the biomarker) are offered currently available treatments at a cost of $23/month (2013 US dollars)^[[8]](#footnote-8)^ (including both patient and insurance spending).^25^
   2. Outcomes are improved and the progression of DKD is slowed in treated individuals. Specifically:
   - Relative risk of kidney disease progression is 0.80 with treatment (with ACE inhibitors or ARBs) compared to without treatment (i.e. risk cut by 20% with treatment)^[[9]](#footnote-9)^.^11^
   - Mortality risk, utility, productivity, and healthcare spending are on average better before DKD progresses to stage 5 CKD than after, so slowed progression equates to improved outcomes.

### Additional scenarios: Combining the biomarker with more effective treatment

Additionally, we ran two scenarios in which we explored how the effects of the biomarker would change if it were combined with more effective treatment for DKD that delays progression to stage 5 CKD. Under these scenarios, we compared results in medical expenditures, QALYs, survival gains, employment, disability, and earnings to the biomarker scenario and to the status quo scenario in which we assumed no intervention.

To model this scenario, we assumed the following.

1. The more effective treatment slows progression to stage 5 CKD more so than current treatment.
   - Risk of kidney disease progression is reduced by an additional 50% or 100% beyond standard of care; i.e. currently available treatment reduces progression risk by 20%,^33^ while the addition of the more effective treatment reduces progression risk by 30% or 40% relative to no treatment.
   - Because outcomes deteriorate with stage 5 CKD, delaying progression translates to improved outcomes

Because this scenario aimed to isolate the effect of the treatment effectiveness on outcomes, we did not change any other assumptions in this scenario, including the treatment cost. Otherwise, we adopted all assumptions from the biomarker scenario.

## Discounting

All net present value costs and benefits were discounted by 3% per year to 2015 dollars.

## Costs

Medical and pharmacy costs were estimated using models based on the MCBS and MEPS and the medical spending growth assumptions detailed above. Additionally, pension, disability, and SSI costs were included based on current claiming and benefit rules.

In addition to a measure of total expenditure, costs can be attributed to Medicaid; Medicare Parts A, B, and D; or out-of-pocket. Commercial payer costs are not currently available.

## Modeling cycle

Modeling cycles in THEMIS are two years in duration.

## Results stratification

For ease of understanding how the flow of patients into DKD and stage 5 CKD affects the study results, results are often stratified into the following groups:

- - Diabetes, no DKD: Individuals with diabetes who do not have DKD
  - DKD: Individuals with DKD. Note that this group includes individuals with stage 5 CKD. Both diagnosed and undiagnosed individuals with DKD are included in this group.
  - Diagnosed DKD: Individuals with a diagnosis of DKD.

Note that individuals may progress over their lifetimes. For example, an individual may begin with diabetes but no DKD, develop (undiagnosed) DKD, then acquire a diagnosis of DKD, and finally progress to stage 5 CKD. These groups refer to all individuals that fall within a given group in a given year. Because study results sometimes vary from year to year, in some cases, results are averaged across multiple years to represent the long-term term trend. Such averaging is noted in the results and exhibits when applicable.

# References

1. Lakdawalla DN, Bhattacharya J, Goldman DP, Hurd M, Joyce GF, Panis CWA. Forecasting the Nursing Home Population. *Medical Care.* 2003;41(1):8-20.

2. Chernew ME, Goldman DP, Pan F, Shang B. Disability and health care spending among medicare beneficiaries. *Health affairs.* 2005;24 Suppl 2:W5R42-52.

3. Michaud PC, Goldman D, Lakdawalla D, Gailey A, Zheng Y. Differences in health between Americans and Western Europeans: Effects on longevity and public finance. *Social Science and Medicine.* 2011;73(2):254-263.

4. Lakdawalla DN, Goldman DP, Shang B. The health and cost consequences of obesity among the future elderly. *Health Aff.* 2005;24(2):W5R30-41.

5. Bhattacharya J, Cutler D, Goldman DP, et al. Disability Forecasts and Future Medicare Costs. In: Cutler D, Garber AM, eds. *Frontiers in Health Policy Research.* Vol 7. Cambridge, MA: MIT Press; 2004:75-94.

6. Goldman DP, Zheng Y, Girosi F, et al. The benefits of risk factor prevention in Americans aged 51 years and older. *Am J Public Health.* 2009;99(11):2096-2101.

7. Goldman DP, Cutler DM, Shang B, Joyce GF. The value of elderly disease prevention. *Forum for Health Economics & Policy.* 2006;9(2).

8. Gaudette E, Messali A, Agus D, Goldman DP. The Long-Term Benefits of Risk Prevention In The United States Elderly. Paper presented at: The Allied Social Science Associations Annual Program; January 3-5, 2016; San Francisco, CA.

9. Congressional Budget Office. *CBO's Long-Term Model: An Overview* 2009.

10. Kumar S, Bogle R, Banerjee D. Why do young people with chronic kidney disease die early? *World J Nephrol.* 2014;3(4):143-155.

11. Kidney International. KDIGO Clinical Practice Guideline for the Evaluation and Management of Chronic Kidney Disease. *Official Journal of the International Society of Nephrology.* 2012;2(1).

12. Naim A, DiBonaventura M, Wagner S, Piech C. Assessing work productivity loss and disability among chronic kidney disease sufferers in the United States. American Occupational Health Conference; 2010; Orlando, FL, USA.

13. de Abreu MM, Walker DR, Sesso RC, Ferraz MB. A cost evaluation of peritoneal dialysis and hemodialysis in the treatment of end-stage renal disease in Sao Paulo, Brazil. *Perit Dial Int.* 2013;33(3):304-315.

14. Zelmer JL. The economic burden of end-stage renal disease in Canada. *Kidney Int.* 2007;72(9):1122-1129.

15. Eggers PW. Medicare's End Stage Renal Disease Program. *Health Care Financing Review.* 2000;22(1):55-60.

16. United States Renal Data System. *2016 USRDS Annual Data Report: Epidemiology of Kidney Disease in the United States.* 2016.

17. Macisaac RJ, Ekinci EI, Jerums G. Markers of and risk factors for the development and progression of diabetic kidney disease. *American journal of kidney diseases : the official journal of the National Kidney Foundation.* 2014;63(2 Suppl 2):S39-62.

18. Gaballa MR, Farag YMK. Predictors of diabetic nephropathy. *Central European Journal of Medicine.* 2012;8(3):287-296.

19. Eknoyan G. Obesity, diabetes, and chronic kidney disease. *Current Diabetes Reports.* 2008;7(6):449-453.

20. Coresh J, Selvin E, Stevens LA, et al. Prevalence of chronic kidney disease in the United States. *JAMA.* 2007;298(17):2038-2047.

21. Chavan VU, Sayyed AK, Durgawale PP, Sontakke AV, Nilakhe SD. Practical Aspects of Calculation, Expression and Interpretation Of Urine Albumin Measurement. *National Journal of Integrated Research in Medicine.* 2011;2(1):29-34.

22. National Kidney Foundation. GFR calculator. 2015; <https://www.kidney.org/professionals/KDOQI/gfr_calculator>. Accessed December 14, 2015.

23. National Kidney Foundation. CKD-EPI Creatinine (2009). 2015; <https://www.kidney.org/content/ckd-epi-creatinine-equation-2009>. Accessed December 14, 2015.

24. National Institute of Diabetes and Digestive Kidney Disease. Estimating Glomerular Filtration Rate (GFR). 2015; <https://www.niddk.nih.gov/health-information/health-communication-programs/nkdep/lab-evaluation/gfr/estimating/Pages/estimating.aspx>. Accessed 4/25/2017.

25. Wetmore J, Li S, Ton T, et al. Elevated risk of end-stage renal disease (ESRD), cardiovascular events, and infection associated with diabetic kidney disease (DKD). *Diabetes.* 2016;66(suppl. 1):A438.

26. National Institute of Diabetes and Digestive Kidney Disease. Kidney disease statistics for the united states. 2012; <https://www.niddk.nih.gov/health-information/health-statistics/Pages/kidney-disease-statistics-united-states.aspx>. Accessed October, 2016.

27. Fox CS, Matsushita K, Woodward M, et al. Associations of kidney disease measures with mortality and end-stage renal disease in individuals with and without diabetes: a meta-analysis. *Lancet (London, England).* 2012;380(9854):1662-1673.

28. Afkarian M, Sachs M, Kestenbaum B, et al. Kidney disease and increased mortality risk in type 2 diabetes. *J Am Soc Nephrol.* 2013;24(2):302-308.

29. Hayes A AH, Woodward M, Chalmers J, Poulter N, Hamet P, Clarke P. Changes in Quality of Life Associated with Complications of Diabetes: Results from the ADVANCE Study. *Value in Health.* 2016;19(1):36-41.

30. United States Renal Data System. *Annual Data Report 2015: Epidemiology of Kidney Disease in the United States* Bethesda, MD: National Institutes of Health, National Institute of Diabetes and Digestive and Kidney Diseases;2015.

31. Muehrer R, Schatell D, Witten B, Gangnon R, Becker B, Hofmann R. Factors affecting employment at initiation of dialysis. *Clin J Am Soc Nephrol.* 2011;6(3):489-496.

32. Niewczas MA, Gohda T, Skupien J, et al. Circulating TNF receptors 1 and 2 predict ESRD in type 2 diabetes. *Journal of the American Society of Nephrology.* 2012;23(3):507-515.

33. Lewis E, Hunsicker L, Clarke W, et al. Renoprotective effect of the angiotensin-receptor antagonist irbesartan in patients with nephropathy due to type 2 diabetes. *N Engl J Med.* 2001;345(12):851-860.

1. For the purposes of the study model, we treated diabetes with comorbid CKD interchangeably with DKD. [↑](#footnote-ref-1)
2. Relative risk of 2.68 was computed using 7.7% 10-year all-cause mortality risk for people without diabetes and without kidney disease (reference group), increased by 3.9% points to 11.6% in adjusted model 1 for people with diabetes and no kidney disease; increased by 23.4% points to 31.1% for people with diabetes and kidney disease. 31.1/11.6 = 2.68. [↑](#footnote-ref-2)
3. Hazard ratio of 4.46 compares the risk of all-cause mortality in individuals with diabetes and eGFR<15 to that of individuals with diabetes and eGFR of 90-104. [↑](#footnote-ref-3)
4. Healthcare cost increase for patients with non-stage 5 CKD DKD was calculated as $24,916 annual Medicare fee-for-service cost for patients with diabetes and (non-stage 5 CKD) kidney disease divided by of $15,718 for patients with diabetes and no kidney disease, giving a 59% increase. [↑](#footnote-ref-4)
5. Healthcare cost increase for patients with diabetes and STAGE 5 CKD was calculated as an annual Medicare cost of $72,653 for patients with stage 5 CKD divided by $15,718 for patients with diabetes, giving a 362% increase. 2013 figures used. [↑](#footnote-ref-5)
6. Increase in likelihood of not working was calculated as 71% unemployment rate among people with stage 5 CKD compared with 9.9% among general population. [↑](#footnote-ref-6)
7. It is assumed that the biomarker test will be conducted at an existing healthcare encounter. [↑](#footnote-ref-7)
8. Based on the average monthly cost of ACE/ARB use among DKD patients in Truven data, according to the companion study [↑](#footnote-ref-8)
9. This relative risk reduction is based on a study of irbesartan compared to placebo (composite endpoint of doubling of serum creatinine, development of stage 5 CKD, or all-cause mortality; 20% risk reduction compared to placebo, p=0.02).[33. Lewis E, Hunsicker L, Clarke W, et al. Renoprotective effect of the angiotensin-receptor antagonist irbesartan in patients with nephropathy due to type 2 diabetes. *N Engl J Med.* 2001;345(12):851-860. [↑](#footnote-ref-9)
